# Microglial heterogeneity: influence of human 2D, 3D, and co-culture models on gene expression and immune function

**DOI:** 10.3389/fncel.2026.1770518

**Published:** 2026-02-11

**Authors:** Fazeleh Etebar, Anthony R. White, Hazel Quek

**Affiliations:** 1 Brain and Mental Health Program, Cellular and Molecular Neurodegeneration, QIMR Berghofer Medical Research Institute, Brisbane, QLD, Australia; 2 School of Biomedical Sciences, Queensland University of Technology, Brisbane, QLD, Australia; 3 School of Biomedical Sciences, The University of Queensland, Brisbane, QLD, Australia; 4 A.I. Virtanen Institute for Molecular Sciences, University of Eastern Finland, Kuopio, Finland; 5 UQ Centre for Clinical Research, The University of Queensland, Brisbane, QLD, Australia

**Keywords:** human monocyte-derived microglia, MDMi, microglial biomarkers, microglial therapeutic targets, neurodegenerative diseases, neuroinflammation, single-cell RNAseq

## Abstract

Microglia, the resident immune cells of the central nervous system, exhibit substantial phenotypic and functional diversity shaped by local microenvironmental cues. While advanced *in vitro* human microglial models exist, the influence of culture dimensionality and cellular context on microglial state composition remains poorly defined. Here, we analyzed single-cell RNA sequencing datasets from human monocyte-derived microglia (MDMi) cultured under two-dimensional (2D) and three-dimensional (3D) monoculture, as well as 3D neural–glial co-culture conditions. Across platforms, four microglial states were identified, including interferon (IFN)-responsive, chemokine-enriched, metabolically active, and proliferative states, with pronounced environment-dependent transcriptional shifts. 2D cultures were dominated by *IFN*-responsive microglia characterized by elevated IFITM2 and IFITM3 expression, whereas 3D systems supported greater cellular diversity, including expanded metabolic programs and chemokine remodeling. Co-culture further increased proliferative microglia and induced immune-communication signatures involving *CCL2/CCL5/CCL7*, *CSF1*, and *VEGF/FLT1* pathways. Pseudotime analysis revealed a largely linear trajectory in 2D cultures, but branching differentiation paths in 3D and co-culture systems, consistent with enhanced microglial heterogeneity. Benchmarking against human microglial reference signatures demonstrated broader and stronger overlap in 3D-based models, with homeostatic and disease-associated modules engaged in a context-specific manner. These findings demonstrate that culture architecture is a major determinant of microglial identity and immune responsiveness; and highlight the value of single-cell datasets to uncover previously underappreciated microglial states with relevance to human neuroimmune biology.

## 1 Introduction

Chronic neuroinflammation, driven largely by microglia, plays a pivotal role in the progression of neurodegenerative diseases such as Alzheimer’s disease (AD), Parkinson’s disease (PD), and amyotrophic lateral sclerosis (ALS). As the resident immune cells of the central nervous system (CNS), microglia exhibit significant phenotypic and functional diversity (Hammond et al., 2019; Masuda et al., 2019; Ochocka et al., 2021; Paolicelli et al., 2022), adopting context-dependent states that can exert neuroprotective or neurotoxic effects depending on disease stage and microenvironmental cues (Michell-Robinson et al., 2015, Colonna and Butovsky, 2017; Friedman et al., 2018; Lloyd et al., 2024). Despite increasing knowledge of microglia’s role in these conditions, accurately modeling this diversity *in vitro* remains a challenge due to the limitations of current culture systems.

Conventional two-dimensional (2D) culture systems are widely used due to their simplicity and scalability; yet they impose physical constraints that alter metabolic state, functional behavior and give rise to transcriptionally distinct sub-populations (Cadiz et al., 2022; Cuní-López et al., 2023). The lack of extracellular matrix architecture and spatial organization limits cell–cell communication and limits the development of microglial transcription states associated with tissue context. In contrast, three-dimensional (3D) cultures better mimic native tissue environments, supporting spatial organization, improved cellular interactions and *in vivo*-like tissue architecture (Shamir and Ewald, 2014; Moysidou et al., 2021; Sharaf et al., 2023). Despite their growing use, the impact of dimensionality alone on microglial identity, subtype composition and differentiation trajectories remain unclear. Recent advances in human microglial modeling have highlighted the importance of environmental context in shaping transcriptional and functional states (Haenseler et al., 2017; Depp et al., 2025; Fumagalli et al., 2025). Microglia exposed to defined cytokine milieus, extracellular matrix properties and cellular neighbors adopt gene expression programs and metabolic profiles that more closely resemble *ex vivo* human microglia (Gosselin et al., 2017; Mancuso et al., 2019; Dolan et al., 2023). 3D culture and co-culture with neural or glial cells promote ramified morphologies, sustained viability and engagement of immune and homeostatic pathways that are poorly captured in 2D monocultures (Schafer et al., 2023; Zhang et al., 2023). Nevertheless, variability in differentiation protocols, matrix composition and co-culture strategies continues to limit cross-study comparability, and systematic evaluations of how dimensionality and cellular context interact to shape microglial differentiation are scarce.

Human primary microglia are difficult to obtain at scale and while induced pluripotent stem cell-derived microglia (iMG) have expanded modeling capacity, differentiation efficiency and maturation state can vary across protocols and donor lines. Monocyte-derived microglia-like cells (MDMi) provide a complementary, accessible human model that retains donor-specific genetic and environmental information, enables longitudinal sampling and is compatible with 3D matrices and multicellular co-culture systems (Ohgidani et al., 2014; Ryan et al., 2017; Cuní-López et al., 2024). Building on prior work establishing patient-derived MDMi platforms (Cuní-López et al., 2024), we reasoned that both culture dimensionality (2D vs. 3D) and cellular context (monoculture versus neural–glial co-culture) would act as key determinants of microglial state composition, maturation dynamics and pathway engagement, even in healthy donor-derived cells.

Here, we leveraged previously characterized MDMi cultured under defined 2D, 3D, and 3D neural-glial co-culture conditions and applied further in-depth analyses to interrogate how microglia adapt to distinct *in vitro* environments. By mapping transcriptional states and differentiation trajectories across culture platforms, we aimed to define how dimensionality and cellular context shape microglial identity at baseline. This framework informs the selection of human microglial models for mechanistic studies, disease modeling and therapeutic discovery.

## 2 Materials and methods

### 2.1 Monocyte-derived microglia-like cell generation and culture conditions

Human monocyte-derived microglia-like cells (MDMi) were generated from peripheral blood monocytes as previously described (Quek et al., 2022; Cuní-López et al., 2024). Briefly, monocytes isolated from healthy donor peripheral blood were differentiated in RPMI-1640 medium supplemented 10 ng/mL GM-CSF and 100 ng/mL IL-34 and maintained under two-dimensional (2D) monoculture, three-dimensional (3D) monoculture or 3D neural–glial co-culture conditions. In 3D cultures, cells were embedded within a Matrigel matrix to provide spatial context, while 3D co-cultures incorporated ReNcell VM–derived neural components that differentiate into mixed neuronal and glial populations.

Prior to single-cell RNA sequencing, cells were dissociated using a non-enzymatic detachment method using a cell recovery solution (Corning, 354253) and fluorescence-activated cell sorting (FACS) was performed to isolate CD11b^+^ cells, thereby excluding leukocytes, neural and glial components. Sorted cells were immediately fixed and processed using the Chromium Next GEM Single Cell Fixed RNA Kit (10x Genomics) according to the manufacturer’s instructions. For scRNA-seq, MDMi were harvested at day 14 in 2D monoculture and at approximately day 30–35 in 3D monoculture and 3D neural–glial co-culture conditions, consistent with the experimental timelines used to generate the GSE255718 dataset (Cuní-López et al., 2024). Detailed experimental protocols are provided in the referenced publication, and no modifications were introduced for the present analysis.

### 2.2 Single-cell RNA sequencing data resource, processing, QC metrics, and clustering

Single-cell RNA sequencing libraries were generated as described previously (Cuní-López et al., 2024). Briefly, libraries were prepared using the Chromium Next GEM Single Cell Fixed RNA Kit (10x Genomics) and sequenced on an Illumina NextSeq 2000 platform. Raw sequencing data were processed using Cell Ranger v7.0.0 (10x Genomics) (Zheng et al., 2017), including demultiplexing, alignment to the human reference genome, and generation of gene–cell count matrices. For the present study, Cell Ranger–generated count matrices were used as the starting point for all downstream analyses. Filtered feature–barcode matrices were imported into R (v4.0.5) (R Core Team, 2019) using the Read10X function and assembled into Seurat objects using CreateSeuratObject (Hao et al., 2021).

Raw gene expression matrices were processed using Seurat. Cells were filtered based on standard quality control metrics, including the number of detected genes per cell, total UMI counts, and the proportion of mitochondrial transcripts. Cells with fewer than 200 detected genes or more than 6,000 detected genes were excluded to remove low-quality cells and potential doublets. Quality control thresholds were applied uniformly across all samples to minimize systematic bias between experimental groups. Following quality control, 12,244 monocytes (from 12,248), 6,472 2D MDMi (from 7,003), 7,736 3D MDMi (from 7,838), and 6,211 3D co-culture MDMi (from 6,426) were retained for downstream analyses (Supplementary Table 1).

Gene expression values were log-normalized and highly variable genes were identified using Seurat’s standard workflow. Data were scaled to minimize the influence of highly expressed genes prior to dimensionality reduction. Principal component analysis (PCA) was performed, followed by non-linear embedding using Uniform Manifold Approximation and Projection (UMAP) or t-distributed stochastic neighbor embedding (*t*-SNE) for visualization. Graph-based clustering was conducted using Seurat’s FindClusters function, with optimal resolution determined using the Clustree package (Zappia and Oshlack, 2018) to avoid over- or under-clustering. Individual samples were processed independently through quality control and normalization prior to aggregation for downstream analyses. Samples were subsequently aggregated using Seurat’s merge() function while retaining sample identity metadata (orig.ident) to enable assessment of potential sample-driven effects.

### 2.3 Identifying differentially expressed genes, cell subtypes and pathway enrichment

Differentially expressed genes (DEGs) between clusters within each sample were identified using Seurat’s ‘FindMarkers’ function with the default Wilcoxon rank-sum test for pairwise comparisons. Resulting *p*-values were adjusted for multiple testing using the Benjamini–Hochberg false discovery rate (FDR) correction. Significantly differentially expressed genes were defined as having an adjusted *p* < 0.05) and log FC > 0.5. Each transcriptionally distinct cluster was subsequently examined to define microglial subtypes based on the expression of canonical marker genes and functional gene signatures. All quantitative differential gene expression statistics underlying dot plots, heat maps and volcano plots are provided in Supplementary Tables 2–5 to enable direct numerical comparison independent of visual scaling.

To gain biological insight into the molecular/biological functions of each subtype, DEGs were analyzed for functional enrichment in pathways and Gene Ontology (GO) terms. Enrichment analysis was performed using an overrepresentation test implemented in the package clusterProfiler (Wu et al., 2021), with significance determined by adjusted *p* < 0.05. Annotation and mapping of gene identifiers (e.g., Entrez ID, Ensembl ID, gene description) were carried out using the AnnotationHub package (Morgan and Shepherd, 2017).

### 2.4 Sample aggregation and pseudotime trajectory mapping of microglial states

Individual samples were processed independently through quality control and normalization and subsequently aggregated using Seurat’s merge() function while retaining sample identity metadata. Because all samples were generated using the same platform, chemistry, and processing pipeline and were processed in parallel, no strong technical batch effects were detected. Data integration methods were therefore not applied, as they may remove genuine biological variability, particularly in highly plastic immune cell populations (Luecken and Theis, 2019; Stuart et al., 2019; Marsh et al., 2022). To examine transition between microglial states and influence of 2D and 3D culture conditions, aggregated scRNA-seq data were used for comparative analysis. Filtered Seurat objects were merged using the merge function, ensuring each cell retained its corresponding sample identifier. Data were normalized using NormalizeData, variable genes were identified via FindVariableFeatures, and expression values were scaled with ScaleData to reduce technical variation across datasets. Dimensionality reduction was performed using PCA (RunPCA), followed by visualization through UMAP and *t*-SNE). All dimensionality reductions were based on the variable features identified during preprocessing. Cluster visualization and sample-specific comparisons were generated using DimPlot, and expression of key markers was examined using FeaturePlot.

To infer dynamic differentiation processes, pseudotime trajectory analysis was conducted using Monocle3 (Trapnell et al., 2014). This analysis enabled reconstruction of developmental trajectories and identification of transcriptional transitions among microglial populations. The integrated Seurat object was converted to a cell_data_set format, and cell clustering and trajectory learning were performed using the cluster_cells and learn_graph functions. The resulting trajectory was visualized with plot_cells, overlaid onto the UMAP representation. A principal node within the monocyte population was designated as the root for pseudotime ordering using order_cells, enabling visualization of temporal progression and branching of microglial states. Cells were color-coded by pseudotime to illustrate differentiation trajectories and state transitions across 2D, 3D and co-culture conditions.

## 3 Results

### 3.1 Four major microglial subtypes identified in 2D and 3D models highlight culture-driven pathway changes

**FIGURE 1:**
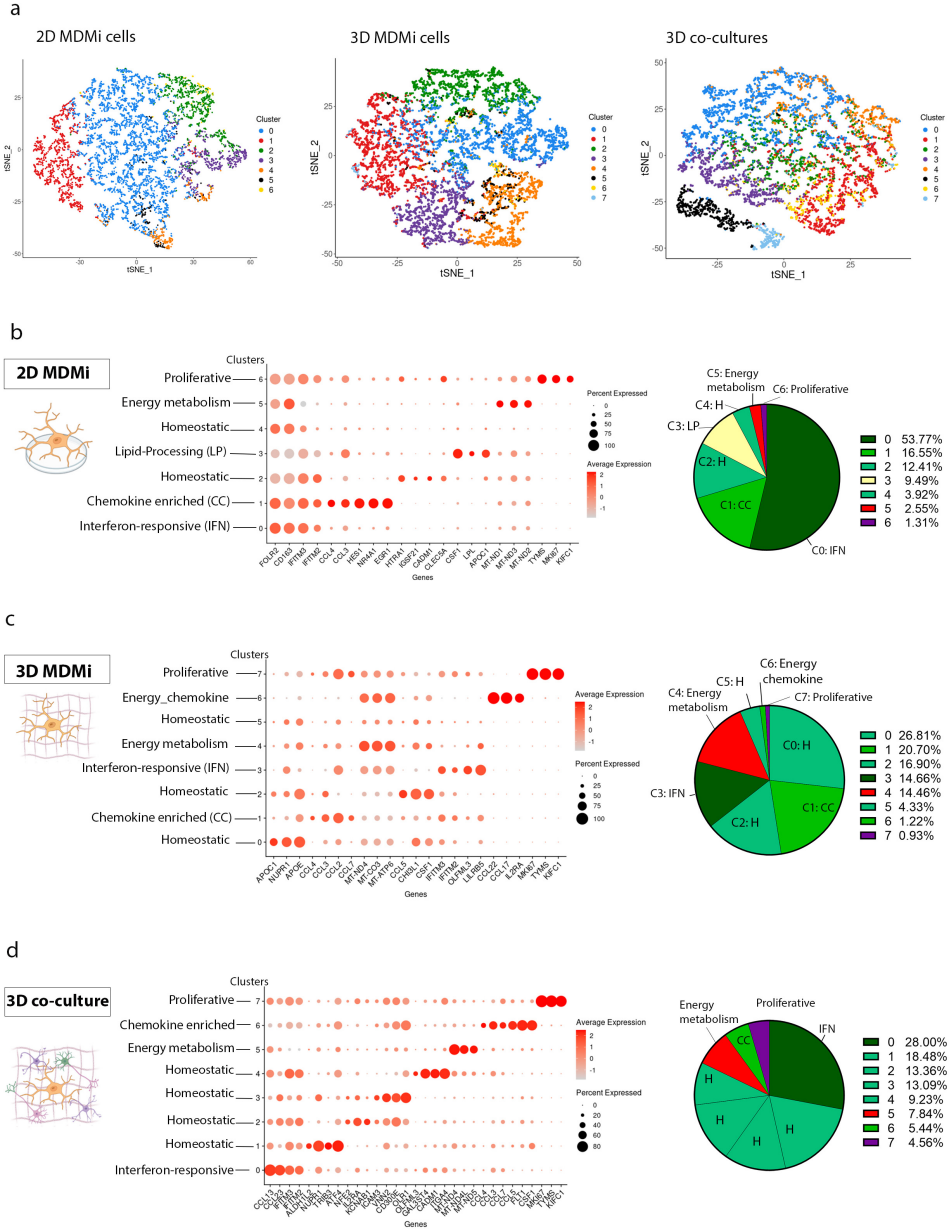
Comparative analysis of microglial subtypes in 2D, 3D, and 3D co-culture models. **(a)** The t-SNE plots depict the clustering of microglial cells based on transcriptomic profiles across the 2D (n = 6,472 cells), 3D (n = 7,736 cells), and 3D co-culture (n = 6,211 cells). Numeric cluster IDs reflect unsupervised clustering performed independently for each culture condition and do not imply correspondence across models; biological cluster identities are defined based on marker gene expression shown in the accompanying dot plots. **(b–d)** Dot plots illustrate gene expression patterns within each identified microglial cluster in 2D, 3D, and 3D co-culture model, with dot size indicating the percentage of cells expressing a given gene and color intensity reflecting the average expression level. Pie charts on the left represent the proportional distribution of the microglial subtypes including proliferative, energy metabolism, chemokine-enriched (CC), and interferon-responsive (IFN) within each culture model.

Single-cell RNA sequencing was performed on CD11b^+^ monocyte-derived microglia generated from healthy donors and cultured under 2D, 3D and 3D neural–glial co-culture conditions, as previously established (Cuní-López et al., 2024). To isolate the effects of culture conditions on microglial transcriptional states, analyses were performed independently of disease context. Using an in-depth, unified clustering and annotation framework, we systematically interrogated microglial heterogeneity across culture environments. Clusters were identified independently for each culture condition using unsupervised clustering (Figure 1a) and annotated *post hoc* based on established microglial marker gene expression (Figures 1b–d). This analysis revealed transcriptional diversity that had not been fully captured previously, identifying four recurrent microglial states including chemokine-enriched (CC), interferon (IFN)-responsive, metabolically active and proliferative, that together comprised the dominant cellular populations across all models (Figures 1a–d). A chemokine-enriched population, characterized by *CCL4* and *CCL3* expression, was consistently observed across all systems, though its abundance and transcriptional composition differed, comprising 16.6% of cells in 2D cultures (Figure 1b), 20.7% in 3D cultures (Figure 1c), and 5.4% in 3D co-cultures (Figure 1d). This cluster displayed environment-specific transcriptional rewiring—in 3D monoculture, cells upregulated *CCL7* and *CCL2*, while the 3D co-culture environment induced expression of *CCL7*, *CCL5*, *FLT1*, and *CSF1*, genes associated with immune cell recruitment, angiogenic signaling and microenvironmental crosstalk. Consistent with prior reports that microglial transcriptional states are highly sensitive to cellular context, we observed environment-dependent changes in chemokine and cell-cell interaction pathways, with co-culture conditions preferentially engaging immune recruitment–associated signaling (e.g., *CCL2/CCL5/CCL7*) (Xue et al., 2021; Gullotta et al., 2023) and microenvironmental/vascular crosstalk (e.g., *CSF1* and *VEGF/FLT1*-associated signaling) (Cakir et al., 2019; Sun et al., 2022). Together, these context-dependent changes illustrate how culture architecture and cellular interaction selectively shape microglial chemokine pathways, revealing transcriptional mechanisms that can confer functional heterogeneity that was obscured in earlier analyses.

Immediate-early genes (IEGs), such as *FOS* and *JUN*, encode AP-1 transcription factors that are rapidly induced via MAPK signaling in response to environmental and immune cues (Shaulian and Karin, 2002). In our dataset, modest enrichment of AP-1/IEG expression in 2D cultures, particularly within cluster 1, is consistent with rigid substrates and limited 3D cell–cell and extracellular matrix interactions, which promote integrin- and MAPK-dependent transcriptional programs (Discher et al., 2005; Glass et al., 2010; Baker and Chen, 2012). Cluster 1 was primarily defined by an interferon-responsive program (*IFITM2*, *IFITM3*, *C1QA–C*), with *FOS* and *JUN* present at low levels and not driving cluster identity, consistent with an immune-alert microglial state rather than dissociation-induced stress (Supplementary Figure 1; Marsh et al., 2022).

The IFN-responsive subtype dominated the 2D model (53.8%), characterized by elevated *IFITM2* and *IFITM3* expression and declined markedly in 3D (14.7%) and 3D co-culture (28%) conditions. Conversely, the 3D model exhibited a more balanced composition of IFN-responsive (14.7%), chemokine-enriched (20.7%) and metabolically active (14.5%) microglia, reflecting greater transcriptional diversity. A metabolically active microglial population was identified across all models, characterized by elevated mitochondrial gene expression, including *MT-ND1*, *MT-ND2* and *MT-ND3*, together with immune-regulatory markers *FOLR2* and *CD163*, indicating increased metabolic activity and energy demand. This population accounted for 2.55% of cells (cluster 5) in 2D culture, 14.46% (cluster 4) and 1.22% (cluster 6) in 3D culture and 7.84% (cluster 5) in 3D co-culture (Figures 1b–d). Within the 3D model, cluster 6 also expressed *CCL22*, *CCL17* and *IL2RA*, consistent with immune-metabolic coupling within the transcriptional profile (Figure 1c).

Interestingly, the 3D co-culture system contained the largest proportion of proliferative microglia (4.56% vs. 1.31% in 2D and 0.93% in 3D), defined by *MKI67*, *TYMS* and *KIFC1* expression, suggesting that the neural environment supports expansion of a cell-cycle-active compartment (Figure 1d). A distinct 2D-specific cluster enriched for *CSF1*, *LPL* and *APOC1* exhibited a lipid-metabolic and phagocytic gene signature, consistent with a transcriptionally activated or disease-associated microglial state (Figure 1b); as previously described in neurodegenerative and inflammatory contexts (Keren-Shaul et al., 2017; Krasemann et al., 2017; Shimizu and Prinz, 2025). Comprehensive expression profiles for all clusters are provided in Supplementary Figures 2–4. Together, these analyses show that microglia acquire distinct transcriptional states across culture environments.

### 3.2 Microglial pathway enrichment patterns differ across culture environments

**FIGURE 2:**
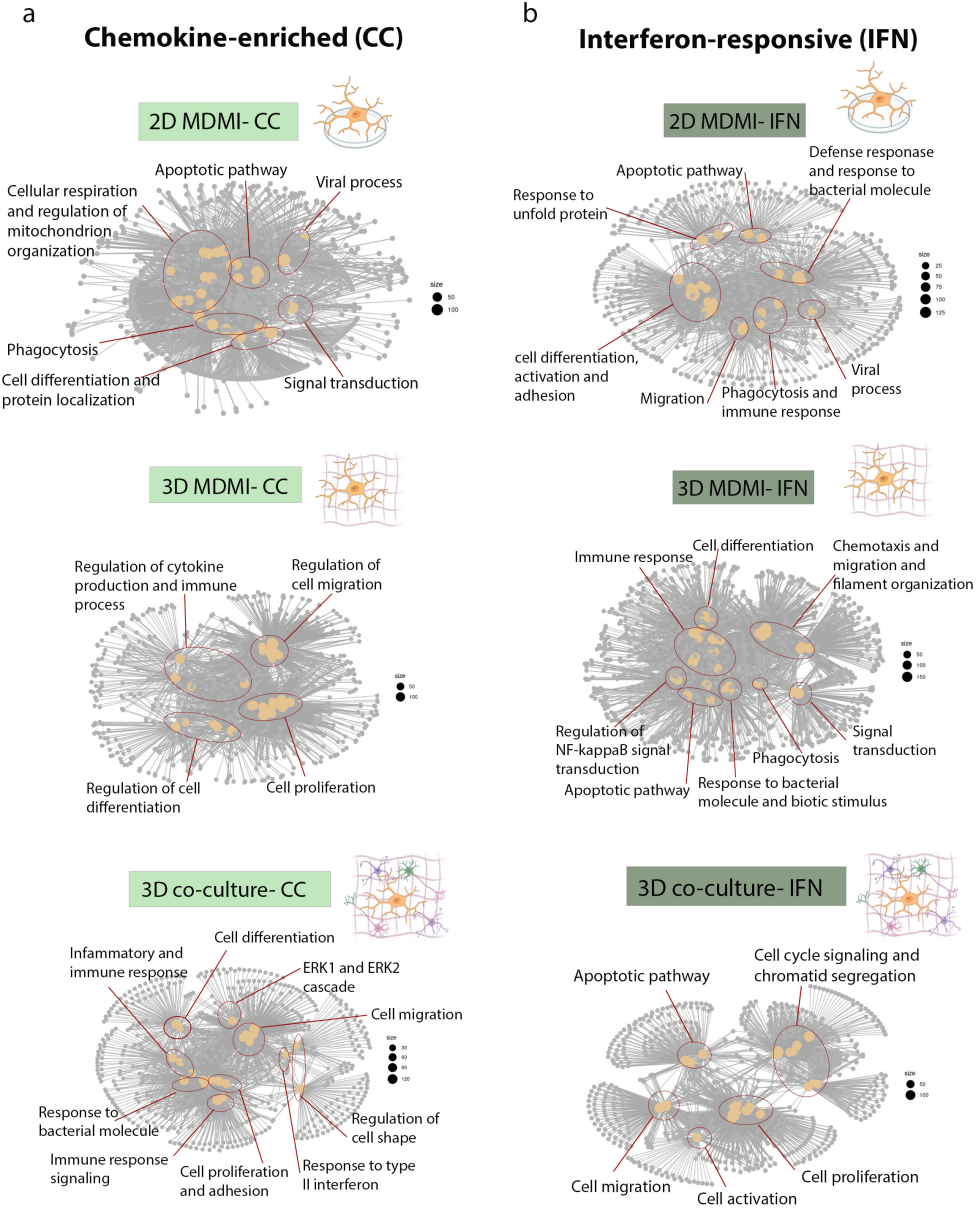
Pathway enrichment networks of chemokine-enriched and interferon-responsive microglial subtypes across culture models. **(a)** The network diagrams illustrate enriched top 30 biological pathways activated in chemokine enriched subtype across models. **(b)** The network diagrams illustrate enriched 30 top biological pathways activated in Interferon responsive subtype across models. Node size reflects the number of genes contributing to each enriched GO term, while edges indicate shared genes between pathways.

To determine how subtype-specific differences translate into broader transcriptional programs, we performed pathway enrichment analysis across 2D, 3D and 3D co-culture microglial models. In 2D culture, chemokine-enriched microglia were associated with pathways governing immune surveillance, including viral responses, phagocytosis, mitochondrial organization and apoptotic regulation. Transitioning to a 3D matrix increased the representation of genes involved in cytokine production, immune activation, immune activation and cellular motility, indicating a transcriptional shift toward immune-signaling pathway. Notably, the 3D co-culture model showed further enrichment of pathways linked to ERK1/ERK2 signaling, bacterial pattern recognition and type II interferon responses. Additional gene set enrichment indicated involvement of pathways regulating cell proliferation, adhesion and differentiation, highlighting transcriptional complexity within the 3D co-culture environment (Figure 2a).

IFN-responsive microglia in 2D culture displayed enrichment for immune defense, phagocytosis and viral response pathways. In 3D conditions, these cells showed increased representation of genes programs linked to chemotaxis, cytoskeletal reorganization, NF-κB signaling and broader inflammatory responses. Within 3D co-cultures, further enrichment of pathways related to cell cycle progression, chromatid segregation and migration reflected activation of proliferative and surveillance-associated gene networks (Figure 2b). Together, these results show that increasing culture complexity (from 2D to 3D and then to co-culture), broadens and strengthens transcriptional programs. We next asked whether these changes alter the core molecular identity of microglia by examining conserved and model-specific gene expression signatures across environments.

### 3.3 Culture environment preserves core microglial identity while shaping transcriptional diversity

**FIGURE 3:**
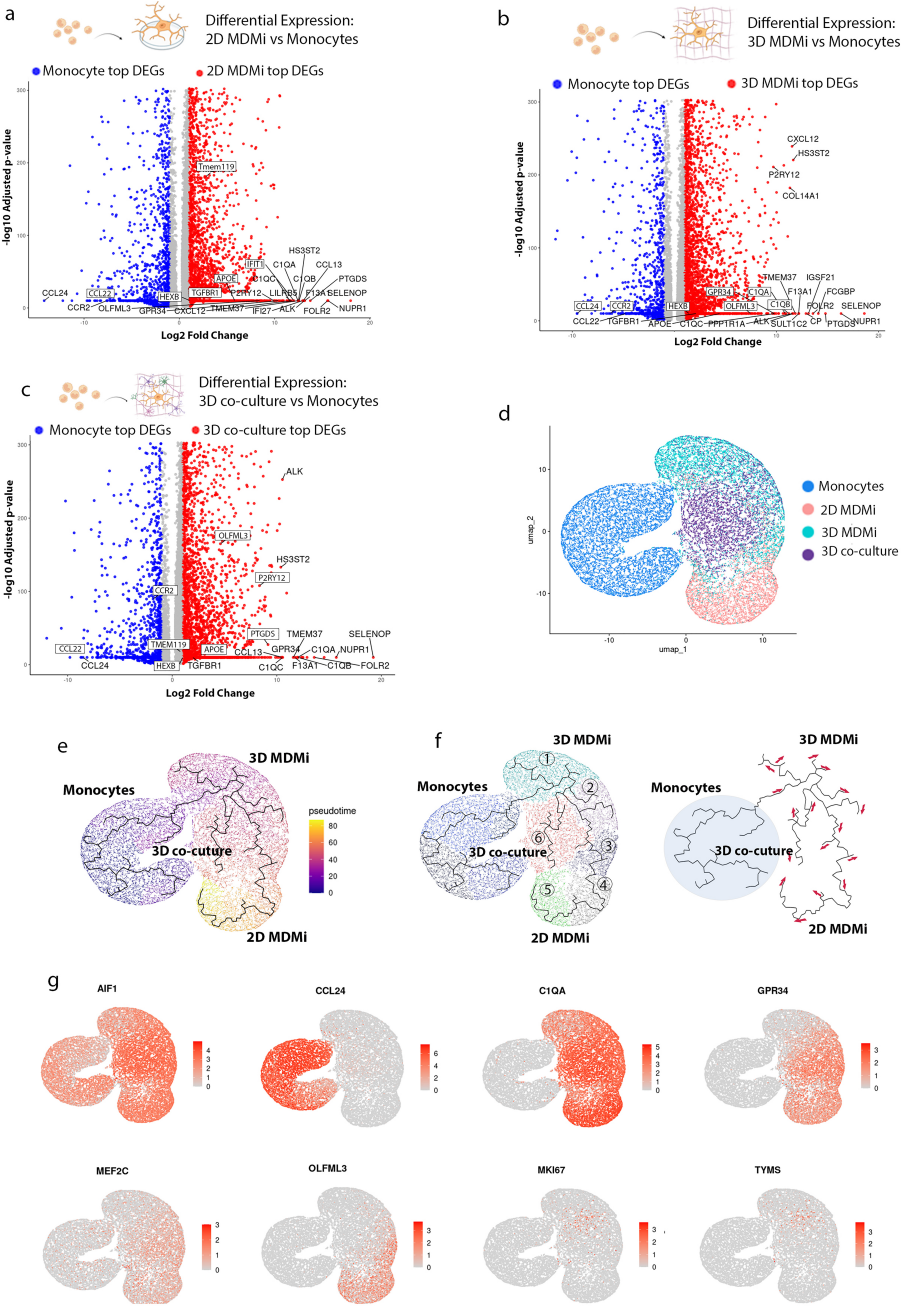
Differential gene expression and pseudotime trajectory of monocyte-to-microglia differentiation across *in vitro* culture models. **(a–c)** Volcano plots illustrating the top upregulated (red) and downregulated (blue) genes in microglia from each model compared to monocytes. **(d)** The UMAP plot illustrates the transcriptomic aggregation of monocytes and microglial cells across the 2D, 3D, and 3D co-culture models. **(e)** UMAP visualization of the pseudotime trajectory depicting monocyte-to-microglia differentiation across the three *in vitro* models. Pseudotime values mapped onto cells, colored from early (dark blue) to late (yellow) differentiation stages. **(f)** Left: UMAP plot with annotated trajectory clusters (1–6), corresponding to distinct phases of differentiation or cellular states. Right: Trajectory structure with directional arrows indicating the progression along pseudotime. **(g)** The UMAPs plots (right) show expression patterns of key genes, including *AIF1*, *CCL24*, *C1QA*, *GPR34*, *MEF2C*, *OLFML3*, *MKI67*, and *TYMS*.

Differentially expressed gene (DEG) analysis revealed that key microglial genes—including *SELENOP*, *NUPR1*, *FOLR2* and *C1QA*—were consistently expressed across 2D, 3D and 3D co-culture systems compared to monocytes, highlighting their central roles in immune regulation and maintenance of microglial identity (Figures 3a–c). This pattern is consistent with prior studies defining these genes as core components of human microglial transcriptional and immune-regulatory programs (Gosselin et al., 2017; Hammond et al., 2019; Masuda et al., 2019). Complement- and chemokine-associated genes such as *C1QB* and *CXCL12* were likewise conserved across 2D and 3D models, indicating preservation of a stable immune-associated transcriptional signature.

The culture environment additionally influenced other transcriptional programs, with 3D conditions promoting increased expression of *FCGBP* and *IGSF21*, genes previously associated with cell–cell communication and extracellular matrix-related processes (Hedegaard et al., 2020). These findings indicate that while the core molecular identity of microglia is largely preserved across models, the magnitude and composition of transcriptional variation are strongly shaped by culture environment.

To explore transcriptional heterogeneity across environments, we applied pseudotime trajectory analysis to integrated single-cell datasets from monocytes, 2D, 3D and 3D co-culture systems (Figure 3d). This approach reconstructed lineage trajectories and captured transitional states across environments. In 2D cultures, cells followed a relatively linear trajectory, consistent with a more uniform transcriptional state. By contrast, 3D and 3D co-cultures exhibited multiple branches, reflecting enhanced transcriptional heterogeneity and the presence of diverse microglial states (Figures 3e,f).

Lineage markers were consistent across trajectories, with sustained expression of the myeloid marker *AIF1* and canonical microglial markers such as *C1QA*, confirming microglial lineage identity in line with prior single-cell studies defining these genes as core components of the microglial transcriptional program (Masuda et al., 2019). In contrast, *CCL24* was enriched at early pseudotime, consistent with progenitor-like or transitional myeloid states as previously reported (Van Hove et al., 2019).

Genes including *GPR34* and *OLFML3* were enriched in distinct trajectory branches, highlighting environment-dependent microglial transcriptional programs (Grabert et al., 2016). Notably, proliferation-associated genes (*TYMS*, *MKI67*) were enriched in terminal branches of the 3D co-culture trajectory, consistent with the emergence of proliferative microglial states in complex, tissue-like conditions (Hammond et al., 2019; Masuda et al., 2019; Figure 3g).

### 3.4 Environmental context drives distinct transcriptional programs in human microglial models

To determine how culture architecture and cellular context shape microglial transcriptional identity, we compared gene expression and pathway activity across MDMi cultured in 2D, 3D monoculture and 3D neural–glial co-culture systems. By integrating differential gene expression and pathway enrichment analyses, we aim to resolve how dimensionality alone, as well as the presence of neural and glial components, influences microglial activation, metabolic and intercellular communication. This unified framework enabled direct comparison of microglial transcriptional programs across progressively complex *in vitro* environments, providing mechanistic insight into how microenvironmental cues drive microglial heterogeneity and putative functional specialization.

**FIGURE 4:**
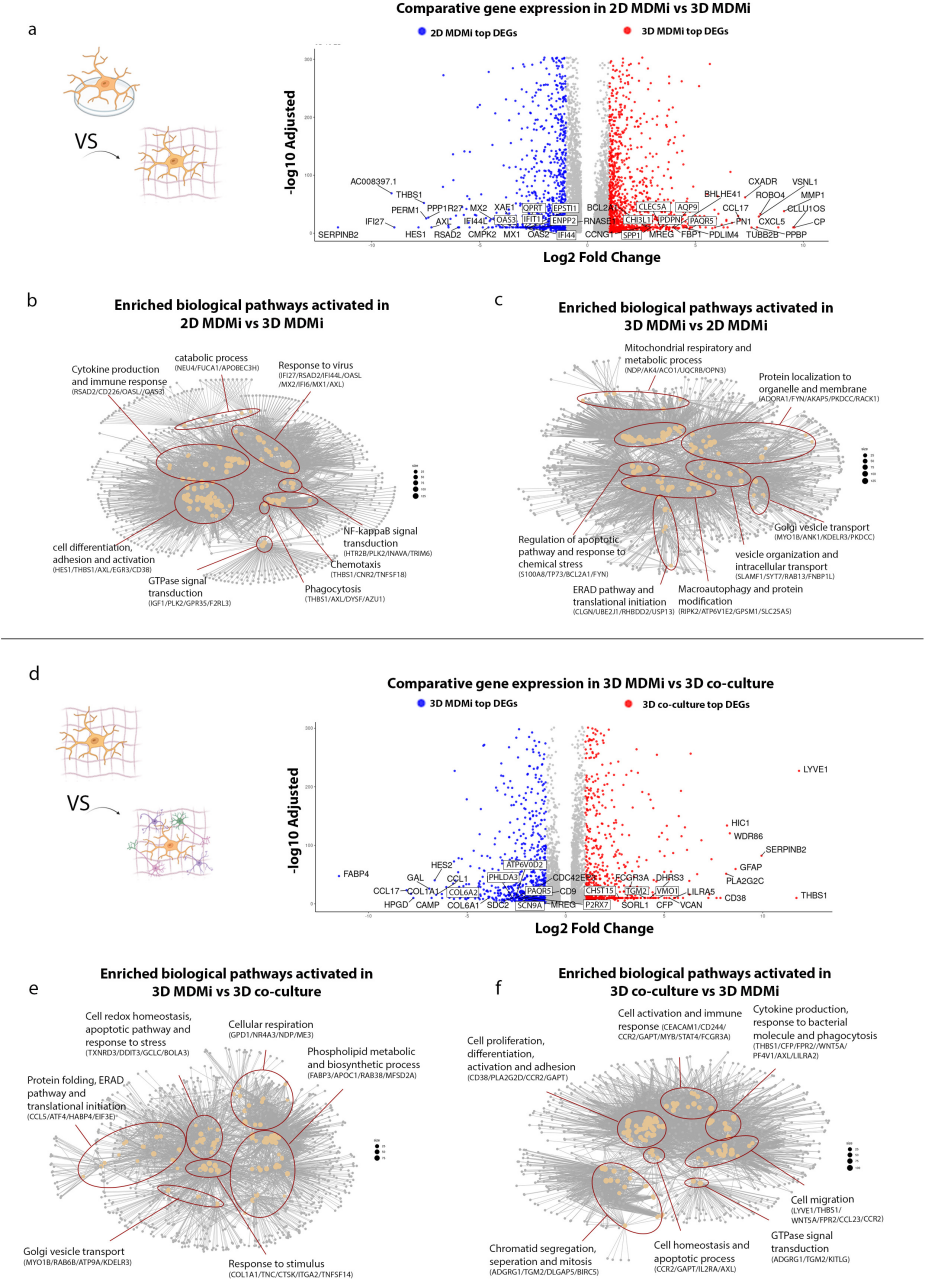
Comparative gene expression and functional pathway analysis between microglial culture models. **(a)** Volcano plot showing differentially expressed genes between 3D and 2D cultures. Genes significantly upregulated in 3D cultures are shown in red, while those upregulated in 2D cultures are shown in blue. **(b)** Network diagram displaying the top 100 enriched biological pathways activated in 2D cultures compared to 3D cultures. **(c)** Network diagram displaying the top 100 enriched biological pathways activated in 3D cultures compared to 2D cultures. **(d)** Volcano plot showing differentially expressed genes between 3D co-culture and 3D monoculture. Genes significantly upregulated in the 3D co-culture are shown in red, while those upregulated in 3D monoculture are shown in blue. **(e)** Network diagram of the top 100 enriched pathways activated in 3D monoculture relative to 3D co-culture. **(f)** Network diagram of the top 100 enriched pathways activated in 3D co-culture relative to 3D monoculture. Genes shown in volcano plots meet differential expression criteria (adjusted *p* < 0.05 and |log2FC| ≥ 0.3); complete DEG statistics and top regulated genes are provided in Supplementary Data. Node size in Network diagrams reflects the number of genes contributing to each enriched GO term, while edges indicate shared genes between pathways.

In 2D cultures, upregulated genes such as *THBS1*, *RSAD2*, *MX2*, and *PERM1* (Figure 4a, blue) were associated with activation of interferon, cytokine and NF-κB–related transcriptional programs, consistent with interferon-responsive microglial signatures observed in single-cell studies of immune-activated states previously reported (Lopez-Atalaya and Bhojwani-Cabrera, 2025; Tang et al., 2025). Pathway enrichment further revealed processes related to cell differentiation and adhesion, suggesting microglial adaptation to 2D culture environment (Figure 4b). In contrast, 3D cultures display pronounced up-regulation of *CXADR*, *ROBO4*, *CCL17*, *CXCL5*, and *MMP1* (Figure 4a, red), indicative of engagement of extracellular-matrix remodeling, immune-cell recruitment and metabolic activation—a pattern aligned with context-dependent microglial transcriptional diversity described in recent reviews of microglia heterogeneity (Fumagalli et al., 2025). Enriched pathways in 3D included mitochondrial respiration, vesicle organization and protein transport, reflecting enhanced transcriptional signatures linked to metabolic and secretory capacity. Collectively, these analyses highlight that 2D cultures preferentially support stress- and immune-primed states, whereas 3D models promote metabolically active and transcriptionally diverse programs, providing a molecular context for the microglial heterogeneity observed in previous analyses (Figure 4c).

Building on the environment-dependent effects, we next compared microglial transcriptional profiles between 3D monoculture and a 3D neural–glial system incorporating ReNcell VM, a human neural progenitor line that spontaneously differentiates into a mixed population of neurons and glial (predominantly astrocytes) (Cuní-López et al., 2024), thereby providing a more complex neural-glial context.

In the 3D monoculture condition, upregulated genes including *FABP4*, *COL1A1*, *CAMP*, and *SDC2* were associated with pathways involved in lipid metabolism and extracellular matrix remodeling (Figure 4d, blue), consistent with previously reported metabolically active or matrix-interacting myeloid and microglial populations (Gosselin et al., 2017; Chia et al., 2025; He et al., 2025). Gene set enrichment further indicated increased representation of transcriptional programs related to cellular respiration, phospholipid biosynthesis and protein folding, consistent with previously described stress-adaptive metabolic states in microglia (Gosselin et al., 2017; Ulland et al., 2017; Figure 4e).

In contrast, microglia within the 3D co-culture system exhibited broader transcriptional diversity, with pronounced enrichment of immune and cell communication–related pathways. Upregulation of *LYVE1*, *SERPINB2*, *HIC1*, and *FCGR3A* pointed to elevated immune responsiveness, phagocytic activity and inflammatory signaling (Figure 4d, red). Expression of *GFAP* and *PLA2G2C* further suggested astrocyte activation and lipid-mediated intercellular signaling, reflecting ongoing neuron-astrocyte-microglia cross-talk. Together, these findings indicate that incorporating ReNcell VM–derived neural components create a more complex transcriptional environment, enhancing immune-related gene expression and intercellular communication compared with 3D monocultures (Figure 4f). Consistent with these environment-dependent transcriptional differences, we also observed model-specific expression of genes that have been previously implicated in neurodegenerative disease pathways, suggesting that distinct culture contexts may differentially engage transcriptional programs that are relevant to, but not specific for, disease-associated states (Supplementary Figure 5).

### 3.5 Benchmarking MDMi clusters against human and mouse microglial reference datasets

We next benchmarked cluster-specific gene signatures from 2D, 3D, and 3D co-culture MDMi against ten independent human microglial reference datasets compiled by Oldham et al. (2008), Miller et al. (2010), Hawrylycz et al. (2012), Darmanis et al. (2015), Wehrspaun et al. (2015), Zhang et al. (2016), Galatro et al. (2017), Gosselin et al. (2017), Patir et al. (2019), and Olah et al. (2020). These reference lists comprise genes annotated to core human microglial identity and to canonical microglial states, including interferon-responsive, chemokine-enriched, metabolic, proliferative and border-associated–like programs, as defined in prior single-cell studies (Grabert et al., 2016; Hammond et al., 2019; Masuda et al., 2019). Across all culture conditions, the largest overlap was consistently observed with the primary human microglia dataset from Olah et al., with cluster-level overlaps ranging from 139–483 genes in 2D, 133–597 genes in 3D and 252–557 genes in 3D co-culture (Supplementary Table 6).

Notably, 3D and 3D co-culture models showed both higher overlap and a broader distribution of overlapping genes across clusters compared with 2D cultures. In 3D MDMi, clusters 2 and 3 each overlapped with more than 500 microglial genes from Olah et al., whereas in 3D co-culture four clusters (1, 3, 4, and 5) overlapped with more than 430 genes. In contrast, 2D clusters showed more restricted overlap, with only clusters 2 and 3 exceeding 450 overlapping genes (Supplementary Tables 6, 7 and Supplementary Figure 6a). These results indicate that 3D-based models capture a larger fraction of established human microglial gene expression.

Given the high overlap with human microglial reference signatures, we next examined the expression of homeostatic and disease-associated microglia (DAM) genes across MDMi clusters (Supplementary Figures 6b–d), using the mouse DAM signature (Keren-Shaul et al., 2017) and recently reviewed (Fumagalli et al., 2025). Genes including *HEXB*, *CST3*, and *C1QA/C1QB* were broadly expressed across clusters in all three culture conditions, indicating stable microglial gene expression across models. By contrast, DAM-associated genes showed heterogeneous, cluster-restricted expression rather than uniform activation, with representative markers such as *TREM2* and *LPL* varying between clusters and culture conditions. Overall, these data show that MDMi preserve key microglial features, and that more complex culture environments drive them into a wider range of microglia-like states that activate established human microglial gene programs.

## 4 Discussion

Microglia display remarkable heterogeneity shaped by developmental origin, regional cues, and environmental context (Yang et al., 2013; Dando et al., 2016, Grabert et al., 2016; De Biase et al., 2017; Ayata et al., 2018; Dubbelaar et al., 2018; Hammond et al., 2019; Marsh et al., 2022; Stogsdill et al., 2022; Yaqubi et al., 2023). To capture this complexity, *in vitro* models have evolved from 2D cultures to iPSC-derived microglia, 3D systems and 3D co-cultures that better reflect human microglial transcriptional signatures and behavior (Park et al., 2018; Liu et al., 2022; Stöberl et al., 2023; Tujula et al., 2025). Building on prior work and our development of patient-derived MDMi systems, this study shows that culture dimensionality and neural context profoundly influence microglial transcriptional profiles, even under homeostatic conditions (Ohgidani et al., 2014; Ryan et al., 2017; Sellgren et al., 2017; Park et al., 2023; Cuní-López et al., 2024; Gonul et al., 2025; Llaves-López et al., 2025; Risby-Jones et al., 2025).

In 2D systems, microglia were dominated by an IFN-responsive state (53.77%), characterized by elevated *IFITM2* and *IFITM3*, markers of antiviral surveillance and innate immune priming (Diamond and Farzan, 2013; Zhou et al., 2013; Fensterl and Sen, 2015). The upregulation of *IFITM3*, a member of the microglial “sensome,” highlights its specialized role in sensing pathogen- and danger-associated molecular patterns (Hickman et al., 2013), and has also been shown to be upregulated in glial cells under AD-related neuroinflammation, supporting its role in mediating immune responses in disease contexts (Hur et al., 2020). The predominance of this state in 2D indicates that the cultures maintain microglia in a more uniform, immune-alert condition, likely reflecting the absence of native extracellular matrix (ECM) cues. In contrast, 3D and co-culture systems diversified the microglial landscape, generating metabolic and proliferative subtypes alongside chemokine-enriched populations. These findings suggest that 3D environments provide spatial and biochemical complexity required to support microglial transcriptional state diversity.

Chemokine-enriched microglia were consistently identified across models but exhibited distinct transcriptional features depending on culture context. In 2D cultures, these microglia expressed antiviral and immune-surveillance genes, whereas in 3D and co-culture systems they upregulated *CCL4*, *CCL3*, *CCL7*, and *CSF1*, consistent with enhanced inflammatory signaling, immune-cell recruitment and matrix interaction. These chemokines have been implicated in neuroinflammation through modulation of BBB integrity and immune cell trafficking (Estevao et al., 2021), highlighting the influence of the microenvironment on immune signaling pathways (Cherry et al., 2020).

Notably, 3D systems contained a higher proportion of metabolically active and proliferative microglia, characterized by increased mitochondrial and biosynthetic gene programs. Although metabolic variation among microglial subtypes remains underexplored, several studies support the influence of local cues on microglial energy-related transcriptional states. For instance, studies have shown that hippocampal and cerebellar microglia exhibit greater expression of metabolic genes than those in cortex and striatum, reflecting region-specific energy demands (Grabert et al., 2016). Similarly, microglia co-culture within human dorsal or ventral forebrain spheroids displays distinct metabolic pathway activity (Song et al., 2019; Zhang et al., 2023). Our findings therefore align with the broader literature indicating that 3D or multicellular contexts promote energy-demanding, adaptive transcriptional programs associated with increased metabolic activity.

Across all models, conserved microglial markers such as *SELENOP*, *NUPR1*, *FOLR2*, *C1QA*, *F13A1*, *ALK*, *CCL13*, *CXCL12*, *C1QB*, *HS3ST2*, and *TMEM37* were consistently expressed, reaffirming their fundamental role in maintaining microglial identity. However, only the 3D and 3D co-culture conditions uniquely induce expression of cell-cell interactions and extracellular matrix components, including *FCGBP* and *IGSF21*, consistent with enhanced cell attachment, growth and differentiation in spatially structured systems (Baker and Chen, 2012; Hedegaard et al., 2020). The 3D co-culture model further distinguished itself by upregulating genes such as *LYVE1*, *SERPINB2*, and *HIC1*, indicative of active cross-talk with neighboring neural and glial cells. These results align with previous findings that 3D co-culture systems recapitulate key tissue-relevant features, including multicellularity and neuronal-glial interactions (Moysidou et al., 2021; Wareham and Calkins, 2025), reinforcing the physiological relevance of this model. Expression of genes such as *LYVE1*, *CD163*, and *CD38* likely reflects the intrinsic plasticity of monocyte-derived microglia and the influence of complex 3D and co-culture environments, which are known to promote macrophage-like, reactive, or inflammatory transcriptional programs. Similar transcriptional states have been reported in *in vitro* microglial and macrophage models exposed to extracellular matrices or inflammatory cues (Wen et al., 2023).

Pseudotime trajectory analysis provided complementary evidence that culture dimensionality influences microglial transcriptional progression and state complexity (Cheng et al., 2023). Microglia in 2D cultures followed a largely linear trajectory consistent with a more constrained and uniform transcriptional profile, whereas 3D and co-culture conditions exhibited branching trajectories, indicating increased transcriptional diversity and alternative state transitions. These patterns are consistent with prior observations that microglial transcriptional states can progress through multiple transitional programs. In 3D, enrichment of genes associated with cytokine signaling, immune pathways and cellular motility was observed, while 3D co-cultures further showed increased representation of ERK1/2 signaling and bacterial recognition-related pathways. Collectively, these findings suggest that 3D monoculture and co-culture systems support more dynamic transcriptional state transitions, providing a framework for modeling context-dependent microglial adaptations observed in neurodegeneration or injury.

Together with prior work, our findings support the use of advanced human *in vitro* systems, particularly 3D and co-culture models, to capture key aspects of microglial biology that are poorly represented in simpler systems. Comparative studies have shown that human primary and iPSC-derived microglia exhibit more robust inflammatory and phagocytic responses than immortalized or murine models (Maguire et al., 2022; Woolf et al., 2025). Consistent with this, MDMi expressed canonical microglial markers absent in monocytes, supporting their utility as an accessible human platform for studying microglia-associated pathways.

### 4.1 Current gaps and outlook

The conclusions of this study apply specifically to monocyte-derived microglia-like cells, and differences in microglial developmental origin are likely to further shape transcriptional identity. While transcriptomic analyses are critical for defining cellular heterogeneity, complementary functional validation is required to link transcriptional diversity to biological outcomes. Functional assays, including phagocytosis, cytokine and chemokine secretion, oxidative stress responses and synaptic pruning, will be necessary to determine whether the microglial states identified here correspond to functionally distinct phenotypes. Integrating such phenotypic readouts with transcriptomic profiling will provide a more comprehensive understanding of microglial behavior across different culture environments. Consistent with this, prior studies have shown that gene expression profiles alone do not fully predict cellular function, underscoring the importance of correlating molecular signatures with functional outputs (Cakir et al., 2022).

## Data Availability

The datasets presented in this study can be found in online repositories. The names of the repository/repositories and accession number(s) can be found in the article/Supplementary material.

## Supplementary material

Supplementary Figure 1 Immediate-early gene expression across culture conditions. **(a)** Dot plot showing expression of immediate-early genes (IEGs) across monocytes, 2D MDMi, 3D MDMi, and 3D MDMi co-cultures. Dot size represents the percentage of cells expressing each gene and color intensity indicates average normalized expression. **(b)** Condition-specific dot plots showing immediate-early gene expression across clusters in 2D MDMi, 3D MDMi, and 3D co-culture conditions.

Supplementary Figure 2 Heatmap of normalized expression for selected differentially expressed genes in each cluster in 2D MDMi model. Range of log2 normalized counts are shown at right hand of heatmap. Blue indicates low expression; and orange indicates high expression.

Supplementary Figure 3 Heatmap of normalized expression for selected differentially expressed genes in each cluster in 3D MDMi model. Range of log2 normalized counts are shown at right hand of heatmap. Blue indicates low expression; and orange indicates high expression.

Supplementary Figure 4 Heatmap of normalized expression for selected differentially expressed genes in each cluster in 3D co-culture. Range of log2 normalized counts are shown at right hand of heatmap. Blue indicates low expression; and orange indicates high expression.

Supplementary Figure 5 Comparative analysis of microglial disease related genes in healthy 2D, 3D, and 3D co-culture models. **(a)** Dot plots illustrate AD related gene expression patterns in microglial models and monocytes, **(b)** dot plots illustrate PD related gene expression patterns in microglial models and monocytes, **(c)** dot plots illustrate ALS related gene expression patterns in microglial models and monocytes. Dot size indicating the percentage of cells expressing a given gene and color intensity reflecting the average expression level.

Supplementary Figure 6 Human microglial signature overlap and marker gene expression across MDMi culture conditions. **(a)** Bar plots showing the number of genes overlapping the human microglial transcriptional signature defined by Olah et al. (2020) across clusters in 2D MDMi, 3D MDMi, and 3D MDMi co-culture conditions. **(b–d)** Violin plots showing expression of selected microglial marker genes across clusters in 2D MDMi **(b)**, 3D MDMi **(c)**, and 3D co-culture **(d)** conditions. Genes are ordered from top to bottom according to canonical microglial identity, homeostatic, and activation-associated DAM genes. Each violin represents the distribution of normalized expression levels within a cluster.

Supplementary Table 1 scRNAseq quality control metrics.

Supplementary Table 2 Shows cluster-specific gene expression in 2D MDMi.

Supplementary Table 3 Shows cluster-specific gene expression in 3D MDMi.

Supplementary Table 4 Shows cluster-specific gene expression in 3D co-culture MDMi.

Supplementary Table 5 Shows DE analysis of all cultures.

Supplementary Table 6 Comparison of MDMi with 10 published human microglia signatures.

Supplementary Table 7 Overlap of cluster-specific MDMi genes with the Olah human microglia gene set.

## References

[B1] Ayata P. Badimon A. Strasburger H. Duff M. Montgomery S. Loh Y. (2018). Epigenetic regulation of brain region-specific microglia clearance activity. *Nat. Neurosci*. 21 1049–1060. 10.1038/s41593-018-0192-3 30038282 PMC6090564

[B2] Baker B. Chen C. (2012). Deconstructing the third dimension: How 3D culture microenvironments alter cellular cues. *J. Cell. Sci.* 125 3015–3024. 10.1242/jcs.079509 22797912 PMC3434846

[B3] Cadiz M. Jensen T. Sens J. Zhu K. Song W. Zhang B. (2022). Culture shock: Microglial heterogeneity, activation, and disrupted single-cell microglial networks in vitro. *Mol. Neurodegener.* 17:26. 10.1186/s13024-022-00531-1 35346293 PMC8962153

[B4] Cakir B. Kiral F. R. Park I.-H. (2022). Advanced in vitro models: Microglia in action. *Neuron* 110 3444–3457. 10.1016/j.neuron.2022.10.004 36327894

[B5] Cakir B. Xiang Y. Tanaka Y. Kural M. Parent M. Kang Y. (2019). Engineering of human brain organoids with a functional vascular-like system. *Nat. Methods* 16 1169–1175. 10.1038/s41592-019-0586-5 31591580 PMC6918722

[B6] Cheng S. Brenière-Letuffe D. Ahola V. Wong A. Keung H. Gurung B. (2023). Single-cell RNA sequencing reveals maturation trajectory in human pluripotent stem cell-derived cardiomyocytes in engineered tissues. *iScience* 26:106302. 10.1016/j.isci.2023.106302 36950112 PMC10025988

[B7] Cherry J. Meng G. Daley S. Xia W. Svirsky S. Alvarez V. (2020). CCL2 is associated with microglia and macrophage recruitment in chronic traumatic encephalopathy. *J. Neuroinflammation* 17:370. 10.1186/s12974-020-02036-4 33278887 PMC7718711

[B8] Chia S. Li M. Li Z. Tu H. Lee J. Qiu L. (2025). Single-nucleus transcriptomics reveals a distinct microglial state and increased MSR1-mediated phagocytosis as common features across dementia subtypes. *Genome Med.* 17:92. 10.1186/s13073-025-01519-4 40826098 PMC12359983

[B9] Colonna M. Butovsky O. (2017). Microglia function in the central nervous system during health and neurodegeneration. *Annu. Rev. Immunol.* 35 441–468. 10.1146/annurev-immunol-051116-052358 28226226 PMC8167938

[B10] Cuní-López C. Stewart R. Oikari L. Nguyen T. Roberts T. Sun Y. (2024). Advanced patient-specific microglia cell models for pre-clinical studies in Alzheimer’s disease. *J. Neuroinflammation* 21:50. 10.1186/s12974-024-03037-3 38365833 PMC10870454

[B11] Cuní-López C. Stewart R. White A. Quek H. (2023). 3D in vitro modelling of human patient microglia: A focus on clinical translation and drug development in neurodegenerative diseases. *J. Neuroimmunol.* 375:578017. 10.1016/j.jneuroim.2023.578017 36657374

[B12] Dando S. Naranjo Golborne C. Chinnery H. Ruitenberg M. McMenamin P. G. A. (2016). case of mistaken identity: cd11c-eyfp(+) cells in the normal mouse brain parenchyma and neural retina display the phenotype of microglia, not dendritic cells. *Glia* 64 1331–1349. 10.1002/glia.23005 27189804

[B13] Darmanis S. Sloan S. Zhang Y. Enge M. Caneda C. Shuer L. (2015). A survey of human brain transcriptome diversity at the single cell level. *Proc. Natl. Acad. Sci. U S A.* 112 7285–7290. 10.1073/pnas.1507125112 26060301 PMC4466750

[B14] De Biase L. Schuebel K. Fusfeld Z. Jair K. Hawes I. Cimbro R. (2017). Local cues establish and maintain region-specific phenotypes of basal ganglia microglia. *Neuron* 95 341–356.e6. 10.1016/j.neuron.2017.06.020 28689984 PMC5754189

[B15] Depp C. Doman J. Hingerl M. Xia J. Stevens B. (2025). Microglia transcriptional states and their functional significance: Context drives diversity. *Immunity* 58 1052–1067. 10.1016/j.immuni.2025.04.009 40328255

[B16] Diamond M. Farzan M. (2013). The broad-spectrum antiviral functions of IFIT and IFITM proteins. *Nat. Rev. Immunol.* 13 46–57. 10.1038/nri3344 23237964 PMC3773942

[B17] Discher D. Janmey P. Wang Y. (2005). Tissue cells feel and respond to the stiffness of their substrate. *Science* 310 1139–1143. 10.1126/science.1116995 16293750

[B18] Dolan M. Therrien M. Jereb S. Kamath T. Gazestani V. Atkeson T. (2023). Exposure of iPSC-derived human microglia to brain substrates enables the generation and manipulation of diverse transcriptional states in vitro. *Nat. Immunol.* 24 1382–1390. 10.1038/s41590-023-01558-2 37500887 PMC10382323

[B19] Dubbelaar M. Kracht L. Eggen B. Boddeke E. (2018). The kaleidoscope of microglial phenotypes. *Front. Immunol.* 9:1753. 10.3389/fimmu.2018.01753 30108586 PMC6079257

[B20] Estevao C. Bowers C. Luo D. Sarker M. Hoeh A. Frudd K. (2021). CCL4 induces inflammatory signalling and barrier disruption in the neurovascular endothelium. *Brain Behav. Immun. Health* 18:100370. 10.1016/j.bbih.2021.100370 34755124 PMC8560974

[B21] Fensterl V. Sen G. (2015). Interferon-induced Ifit proteins: Their role in viral pathogenesis. *J. Virol.* 89 2462–2468. 10.1128/JVI.02744-14 25428874 PMC4325746

[B22] Friedman B. Srinivasan K. Ayalon G. Meilandt W. Lin H. Huntley M. (2018). Diverse brain myeloid expression profiles reveal distinct microglial activation states and aspects of Alzheimer’s disease not evident in mouse models. *Cell. Rep.* 22 832–847. 10.1016/j.celrep.2017.12.066 29346778

[B23] Fumagalli L. Nazlie Mohebiany A. Premereur J. Polanco Miquel P. Bijnens B. Van de Walle P. (2025). Microglia heterogeneity, modeling and cell-state annotation in development and neurodegeneration. *Nat. Neurosci.* 28 1381–1392. 10.1038/s41593-025-01931-4 40195564

[B24] Galatro T. Holtman I. Lerario A. Vainchtein I. Brouwer N. Sola P. (2017). Transcriptomic analysis of purified human cortical microglia reveals age-associated changes. *Nat. Neurosci.* 20 1162–1171. 10.1038/nn.4597 28671693

[B25] Glass C. Saijo K. Winner B. Marchetto M. Gage F. (2010). Mechanisms underlying inflammation in neurodegeneration. *Cell* 140 918–934. 10.1016/j.cell.2010.02.016 20303880 PMC2873093

[B26] Gonul C. Kiser C. Yaka E. Oz D. Hunerli D. Yerlikaya D. (2025). Microglia-like cells from patient monocytes demonstrate increased phagocytic activity in probable Alzheimer’s disease. *Mol. Cell. Neurosci.* 132:103990. 10.1016/j.mcn.2024.103990 39732446

[B27] Gosselin D. Skola D. Coufal N. Holtman I. Schlachetzki J. Sajti E. (2017). An environment-dependent transcriptional network specifies human microglia identity. *Science* 356:eaal3222. 10.1126/science.aal3222 28546318 PMC5858585

[B28] Grabert K. Michoel T. Karavolos M. Clohisey S. Baillie J. Stevens M. (2016). Microglial brain region-dependent diversity and selective regional sensitivities to aging. *Nat. Neurosci.* 19 504–516. 10.1038/nn.4222 26780511 PMC4768346

[B29] Gullotta G. Costantino G. Sortino M. Spampinato S. (2023). Microglia and the blood-brain barrier: An external player in acute and chronic neuroinflammatory conditions. *Int. J. Mol. Sci.* 24:9144. 10.3390/ijms24119144 37298096 PMC10252410

[B30] Haenseler W. Sansom S. Buchrieser J. Newey S. Moore C. Nicholls F. (2017). A highly efficient human pluripotent stem cell microglia model displays a neuronal-co-culture-specific expression profile and inflammatory response. *Stem. Cell. Reports* 8 1727–1742. 10.1016/j.stemcr.2017.05.017 28591653 PMC5470330

[B31] Hammond T. Dufort C. Dissing-Olesen L. Giera S. Young A. Wysoker A. (2019). Single-Cell RNA sequencing of microglia throughout the mouse lifespan and in the injured brain reveals complex cell-state changes. *Immunity* 50 253–271.e6. 10.1016/j.immuni.2018.11.004 30471926 PMC6655561

[B32] Hao Y. Hao S. Andersen-Nissen E. Mauck W. Zheng S. Butler A. (2021). Integrated analysis of multimodal single-cell data. *Cell* 184 3573–3587.e29. 10.1016/j.cell.2021.04.048 34062119 PMC8238499

[B33] Hawrylycz M. Lein E. Guillozet-Bongaarts A. Shen E. Ng L. Miller J. (2012). An anatomically comprehensive atlas of the adult human brain transcriptome. *Nature* 489 391–399. 10.1038/nature11405 22996553 PMC4243026

[B34] He J. Qin Z. Liu H. Cai Y. Wu H. Wang T. (2025). Fatty acid-binding protein 4 drives microglia-mediated neuroinflammation through promoting S100A9 expression and lipid droplet accumulation after intracerebral hemorrhage. *J. Neuroinflammation* 22:263. 10.1186/s12974-025-03573-6 41204337 PMC12595865

[B35] Hedegaard A. Stodolak S. James W. Cowley S. (2020). Honing the double-edged sword: Improving human iPSC-Microglia models. *Front. Immunol.* 11:614972. 10.3389/fimmu.2020.614972 33363548 PMC7753623

[B36] Hickman S. Kingery N. Ohsumi T. Borowsky M. Wang L. Means T. (2013). The microglial sensome revealed by direct RNA sequencing. *Nat. Neurosci.* 16 1896–1905. 10.1038/nn.3554 24162652 PMC3840123

[B37] Hur J. Frost G. Wu X. Crump C. Pan S. Wong E. (2020). The innate immunity protein IFITM3 modulates γ-secretase in Alzheimer’s disease. *Nature* 586 735–740. 10.1038/s41586-020-2681-2 32879487 PMC7919141

[B38] Keren-Shaul H. Spinrad A. Weiner A. Matcovitch-Natan O. Dvir-Szternfeld R. Ulland T. (2017). A unique microglia type associated with restricting development of Alzheimer’s disease. *Cell* 169 1276–1290.e17. 10.1016/j.cell.2017.05.018 28602351

[B39] Krasemann S. Madore C. Cialic R. Baufeld C. Calcagno N. El Fatimy R. (2017). The TREM2-APOE pathway drives the transcriptional phenotype of dysfunctional microglia in neurodegenerative diseases. *Immunity* 47 566–581.e9. 10.1016/j.immuni.2017.08.008 28930663 PMC5719893

[B40] Liu R. Meng X. Yu X. Wang G. Dong Z. Zhou Z. (2022). From 2D to 3D Co-culture systems: A review of co-culture models to study the neural cells interaction. *Int. J. Mol. Sci.* 23:13116. 10.3390/ijms232113116 36361902 PMC9656609

[B41] Llaves-López A. Micoli E. Belmonte-Mateos C. Aguilar G. Alba C. Marsal A. (2025). Human microglia-like cells differentiated from monocytes with GM-CSF and IL-34 show phagocytosis of α-Synuclein aggregates and C/EBPβ-Dependent proinflammatory activation. *Mol. Neurobiol.* 62 756–772. 10.1007/s12035-024-04289-z 38900366 PMC11711251

[B42] Lloyd A. Martinez-Muriana A. Davis E. Daniels M. Hou P. Mancuso R. (2024). Deep proteomic analysis of microglia reveals fundamental biological differences between model systems. *Cell. Rep.* 43:114908. 10.1016/j.celrep.2024.114908 39460937

[B43] Lopez-Atalaya J. Bhojwani-Cabrera A. (2025). Type I interferon signalling and interferon-responsive microglia in health and disease. *FEBS J.* 292 5921–5940. 10.1111/febs.70126 40299722 PMC12631166

[B44] Luecken M. Theis F. (2019). Current best practices in single-cell RNA-seq analysis: A tutorial. *Mol. Syst. Biol.* 15:e8746. 10.15252/msb.20188746 31217225 PMC6582955

[B45] Maguire E. Connor-Robson N. Shaw B. O’Donoghue R. Stöberl N. Hall-Roberts H. (2022). Assaying microglia functions in vitro. *Cells* 11:3414. 10.3390/cells11213414 36359810 PMC9654693

[B46] Mancuso R. Van Den Daele J. Fattorelli N. Wolfs L. Balusu S. Burton O. (2019). Stem-cell-derived human microglia transplanted in mouse brain to study human disease. *Nat. Neurosci.* 22 2111–2116. 10.1038/s41593-019-0525-x 31659342 PMC7616913

[B47] Marsh S. Walker A. Kamath T. Dissing-Olesen L. Hammond T. de Soysa T. (2022). Dissection of artifactual and confounding glial signatures by single-cell sequencing of mouse and human brain. *Nat. Neurosci.* 25 306–316. 10.1038/s41593-022-01022-8 35260865 PMC11645269

[B48] Masuda T. Sankowski R. Staszewski O. Böttcher C. Amann L. Sagar (2019). Spatial and temporal heterogeneity of mouse and human microglia at single-cell resolution. *Nature* 566 388–392. 10.1038/s41586-019-0924-x 30760929

[B49] Michell-Robinson M. Touil H. Healy L. Owen D. Durafourt B. Bar-Or A. (2015). Roles of microglia in brain development, tissue maintenance and repair. *Brain* 138 1138–1159. 10.1093/brain/awv066 25823474 PMC5963417

[B50] Miller J. Horvath S. Geschwind D. (2010). Divergence of human and mouse brain transcriptome highlights Alzheimer disease pathways. *Proc. Natl. Acad. Sci. U S A.* 107 12698–12703. 10.1073/pnas.0914257107 20616000 PMC2906579

[B51] Morgan M. Shepherd L. (2017). *AnnotationHub: Client to Access AnnotationHub resources. R package version 2, no. 1.*

[B52] Moysidou C. Barberio C. Owens R. (2021). Advances in engineering human tissue models. *Front. Bioeng. Biotechnol.* 8:620962. 10.3389/fbioe.2020.620962 33585419 PMC7877542

[B53] Ochocka N. Segit P. Walentynowicz K. Wojnicki K. Cyranowski S. Swatler J. (2021). Single-cell RNA sequencing reveals functional heterogeneity of glioma-associated brain macrophages. *Nat. Commun.* 12:1151. 10.1038/s41467-021-21407-w 33608526 PMC7895824

[B54] Ohgidani M. Kato T. Setoyama D. Sagata N. Hashimoto R. Shigenobu K. (2014). Direct induction of ramified microglia-like cells from human monocytes: Dynamic microglial dysfunction in Nasu-Hakola disease. *Sci. Rep.* 4:4957. 10.1038/srep04957 24825127 PMC4019954

[B55] Olah M. Menon V. Habib N. Taga M. Ma Y. Yung C. (2020). Single cell RNA sequencing of human microglia uncovers a subset associated with Alzheimer’s disease. *Nat. Commun.* 11:6129. 10.1038/s41467-020-19737-2 33257666 PMC7704703

[B56] Oldham M. Konopka G. Iwamoto K. Langfelder P. Kato T. Horvath S. (2008). Functional organization of the transcriptome in human brain. *Nat. Neurosci.* 11 1271–1282. 10.1038/nn.2207 18849986 PMC2756411

[B57] Paolicelli R. C. Sierra A. Stevens B. Tremblay M.-E. Aguzzi A. Ajami B. (2022). Microglia states and nomenclature: A field at its crossroads. *Neuron* 110 3458–3483. 10.1016/j.neuron.2022.10.020 36327895 PMC9999291

[B58] Park D. Kozaki T. Tiwari S. Moreira M. Khalilnezhad A. Torta F. (2023). iPS-cell-derived microglia promote brain organoid maturation via cholesterol transfer. *Nature* 623 397–405. 10.1038/s41586-023-06713-1 37914940

[B59] Park J. Wetzel I. Dréau D. D’Avanzo C. Kim D. (2018). A 3D human triculture system modeling neurodegeneration and neuroinflammation in Alzheimer’s disease. *Nat. Neurosci.* 21 941–951. 10.1038/s41593-018-0175-4 29950669 PMC6800152

[B60] Patir A. Shih B. McColl B. Freeman T. C. A. (2019). core transcriptional signature of human microglia: Derivation and utility in describing region-dependent alterations associated with Alzheimer’s disease. *Glia* 67 1240–1253. 10.1002/glia.23572 30758077

[B61] Quek H. Cuní-López C. Stewart R. Lim Y. Roberts T. White A. R. (2022). A robust approach to differentiate human monocyte-derived microglia from peripheral blood mononuclear cells. *STAR Protoc.* 3:101747. 10.1016/j.xpro.2022.101747 36201317 PMC9535318

[B62] R Core Team. (2019). *R: A Language and Environment for Statistical Computing.* Vienna: R Foundation for Statistical Computing.

[B63] Risby-Jones G. Marallag J. Jagaraj C. Atkin J. Walker A. Woodruff T. (2025). IL-6 trans-signalling is elevated in ALS models and drives TDP-43 induced inflammatory responses in microglia. *Brain Behav. Immun.* 129 296–304. 10.1016/j.bbi.2025.06.021 40523537

[B64] Ryan K. White C. Patel K. Xu J. Olah M. Replogle J. (2017). A human microglia-like cellular model for assessing the effects of neurodegenerative disease gene variants. *Sci. Transl. Med.* 9:eaai7635. 10.1126/scitranslmed.aai7635 29263232 PMC5945290

[B65] Schafer S. Mansour A. Schlachetzki J. Pena M. Ghassemzadeh S. Mitchell L. (2023). An in vivo neuroimmune organoid model to study human microglia phenotypes. *Cell* 186 2111–2126.e20. 10.1016/j.cell.2023.04.022 37172564 PMC10284271

[B66] Sellgren C. Sheridan S. Gracias J. Xuan D. Fu T. Perlis R. (2017). Patient-specific models of microglia-mediated engulfment of synapses and neural progenitors. *Mol. Psychiatry* 22 170–177. 10.1038/mp.2016.220 27956744 PMC5285468

[B67] Shamir E. Ewald A. (2014). Three-dimensional organotypic culture: Experimental models of mammalian biology and disease. *Nat. Rev. Mol. Cell. Biol.* 15 647–664. 10.1038/nrm3873 25237826 PMC4352326

[B68] Sharaf A. Timmerman R. Bajramovic J. Accardo A. (2023). In vitro microglia models: The era of engineered cell microenvironments. *Neural Regen. Res.* 18 1709–1710. 10.4103/1673-5374.363828 36751786 PMC10154482

[B69] Shaulian E. Karin M. (2002). AP-1 as a regulator of cell life and death. *Nat. Cell. Biol.* 4 E131–E136. 10.1038/ncb0502-e131 11988758

[B70] Shimizu T. Prinz M. (2025). Microglia across evolution: From conserved origins to functional divergence. *Cell. Mol. Immunol.* 22 1533–1548. 10.1038/s41423-025-01368-6 41272275 PMC12660708

[B71] Song L. Yuan X. Jones Z. Vied C. Miao Y. Marzano M. (2019). Functionalization of brain region-specific spheroids with isogenic microglia-like cells. *Sci. Rep.* 9:11055. 10.1038/s41598-019-47444-6 31363137 PMC6667451

[B72] Stöberl N. Maguire E. Salis E. Shaw B. Hall-Roberts H. (2023). Human iPSC-derived glia models for the study of neuroinflammation. *J. Neuroinflammation* 20:231. 10.1186/s12974-023-02919-2 37817184 PMC10566197

[B73] Stogsdill J. Kim K. Binan L. Farhi S. Levin J. Arlotta P. (2022). Pyramidal neuron subtype diversity governs microglia states in the neocortex. *Nature* 608 750–756. 10.1038/s41586-022-05056-7 35948630 PMC10502800

[B74] Stuart T. Butler A. Hoffman P. Hafemeister C. Papalexi E. Mauck W. (2019). Comprehensive integration of single-*cell* data. *Cell* 177 1888–1902.e21. 10.1016/j.cell.2019.05.031 31178118 PMC6687398

[B75] Sun X. Ju X. Li Y. Zeng P. Wu J. Zhou Y. (2022). Generation of vascularized brain organoids to study neurovascular interactions. *Elife* 11:e76707. 10.7554/eLife.76707 35506651 PMC9246368

[B76] Tang Y. Zhang Q. Qu Y. Yi L. Li F. Qu C. (2025). Single-cell RNA sequencing reveals microglial heterogeneity and functional states after cerebral ischemia-reperfusion injury. *J. Inflamm. Res.* 18 16931–16956. 10.2147/JIR.S539404 41368344 PMC12684991

[B77] Trapnell C. Cacchiarelli D. Grimsby J. Pokharel P. Li S. Morse M. (2014). The dynamics and regulators of cell fate decisions are revealed by pseudotemporal ordering of single cells. *Nat. Biotechnol.* 32 381–386. 10.1038/nbt.2859 24658644 PMC4122333

[B78] Tujula I. Hyvärinen T. Lotila J. Rogal J. Voulgaris D. Sukki L. (2025). Modeling neuroinflammatory interactions between microglia and astrocytes in a human iPSC-based coculture platform. *Cell Commun. Signal.* 23:298. 10.1186/s12964-025-02304-x 40542355 PMC12181861

[B79] Ulland T. Song W. Huang S. Ulrich J. Sergushichev A. Beatty W. (2017). TREM2 maintains microglial metabolic fitness in Alzheimer’s disease. *Cell* 170 649–663.e13. 10.1016/j.cell.2017.07.023 28802038 PMC5573224

[B80] Van Hove H. Martens L. Scheyltjens I. De Vlaminck K. Pombo Antunes A. De Prijck S. (2019). A single-cell atlas of mouse brain macrophages reveals unique transcriptional identities shaped by ontogeny and tissue environment. *Nat. Neurosci.* 22 1021–1035. 10.1038/s41593-019-0393-4 31061494

[B81] Wareham L. Calkins D. (2025). Making tracks: Microglia and the extracellular matrix. *Mol. Neurodegener.* 20:101. 10.1186/s13024-025-00898-x 41024112 PMC12481997

[B82] Wehrspaun C. Haerty W. Ponting C. (2015). Microglia recapitulate a hematopoietic master regulator network in the aging human frontal cortex. *Neurobiol. Aging* 36 2443.e9–2443.e20. 10.1016/j.neurobiolaging.2015.04.008 26002684 PMC4503803

[B83] Wen W. Cheng J. Tang Y. (2023). *Brain* perivascular macrophages: Current understanding and future prospects. *Brain* 147 39–55. 10.1093/brain/awad304 37691438 PMC10766266

[B84] Woolf Z. Stevenson T. Lee K. Highet B. Macapagal Foliaki J. Ratiu R. (2025). In vitro models of microglia: A comparative study. *Sci. Rep.* 15:15621. 10.1038/s41598-025-99867-z 40320508 PMC12050316

[B85] Wu T. Hu E. Xu S. Chen M. Guo P. Dai Z. (2021). clusterProfiler 4.0: A universal enrichment tool for interpreting omics data. *Innovation* 2:100141. 10.1016/j.xinn.2021.100141 34557778 PMC8454663

[B86] Xue J. Zhang Y. Zhang J. Zhu Z. Lv Q. Su J. (2021). Astrocyte-derived CCL7 promotes microglia-mediated inflammation following traumatic brain injury. *Int. Immunopharmacol.* 99:107975. 10.1016/j.intimp.2021.107975 34293712

[B87] Yang T. Lin C. Hsu C. Wang T. Ke F. Kuo Y. (2013). Differential distribution and activation of microglia in the brain of male C57BL/6J mice. *Brain Struct. Funct.* 218 1051–1060. 10.1007/s00429-012-0446-x 22886465

[B88] Yaqubi M. Groh A. Dorion M. Afanasiev E. Luo J. Hashemi H. (2023). Analysis of the microglia transcriptome across the human lifespan using single cell RNA sequencing. *J. Neuroinflammation* 20:132. 10.1186/s12974-023-02809-7 37254100 PMC10230780

[B89] Zappia L. Oshlack A. (2018). Clustering trees: A visualization for evaluating clusterings at multiple resolutions. *Gigascience* 7:giy083. 10.1093/gigascience/giy083 30010766 PMC6057528

[B90] Zhang W. Jiang J. Xu Z. Yan H. Tang B. Liu C. (2023). Microglia-containing human brain organoids for the study of brain development and pathology. *Mol. Psychiatry* 28 96–107. 10.1038/s41380-022-01892-1 36474001 PMC9734443

[B91] Zhang Y. Sloan S. Clarke L. Caneda C. Plaza C. Blumenthal P. (2016). Purification and characterization of progenitor and mature human astrocytes reveals transcriptional and functional differences with mouse. *Neuron* 89 37–53. 10.1016/j.neuron.2015.11.013 26687838 PMC4707064

[B92] Zheng G. Terry J. Belgrader P. Ryvkin P. Bent Z. Wilson R. (2017). Massively parallel digital transcriptional profiling of single cells. *Nat. Commun.* 8:14049. 10.1038/ncomms14049 28091601 PMC5241818

[B93] Zhou X. Michal J. Zhang L. Ding B. Lunney J. Liu B. (2013). Interferon induced IFIT family genes in host antiviral defense. *Int. J. Biol. Sci.* 9 200–208. 10.7150/ijbs.5613 23459883 PMC3584916

