# Supplementary figures and images for: Microglial heterogeneity: influence of human 2D, 3D, and co-culture models on gene expression and immune function
Source: Front Cell Neurosci. 2026 Feb 11;20:1770518. doi: 10.3389/fncel.2026.1770518 (PMC12932485; doi:10.3389/fncel.2026.1770518)

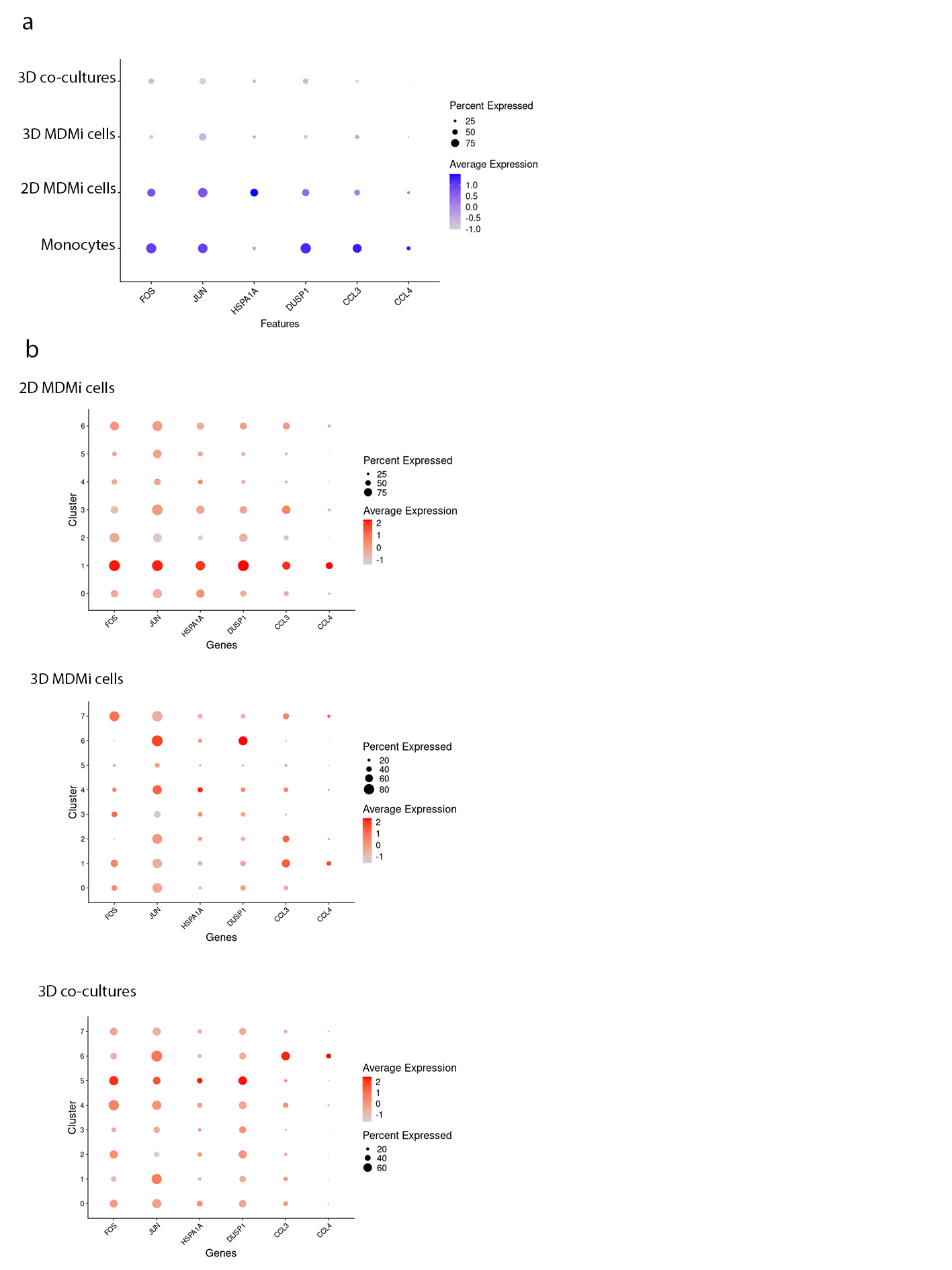

Supplement: Supplementary Figure 1 — Immediate-early gene expression across culture conditions. (a) Dot plot showing expression of immediate-early genes (IEGs) across monocytes, 2D MDMi, 3D MDMi, and 3D MDMi co-cultures. Dot size represents the percentage of cells expressing each gene and color intensity indicates average normalized expression. (b) Condition-specific dot plots showing immediate-early gene expression across clusters in 2D MDMi, 3D MDMi, and 3D co-culture conditions. [file Image_1.tif]

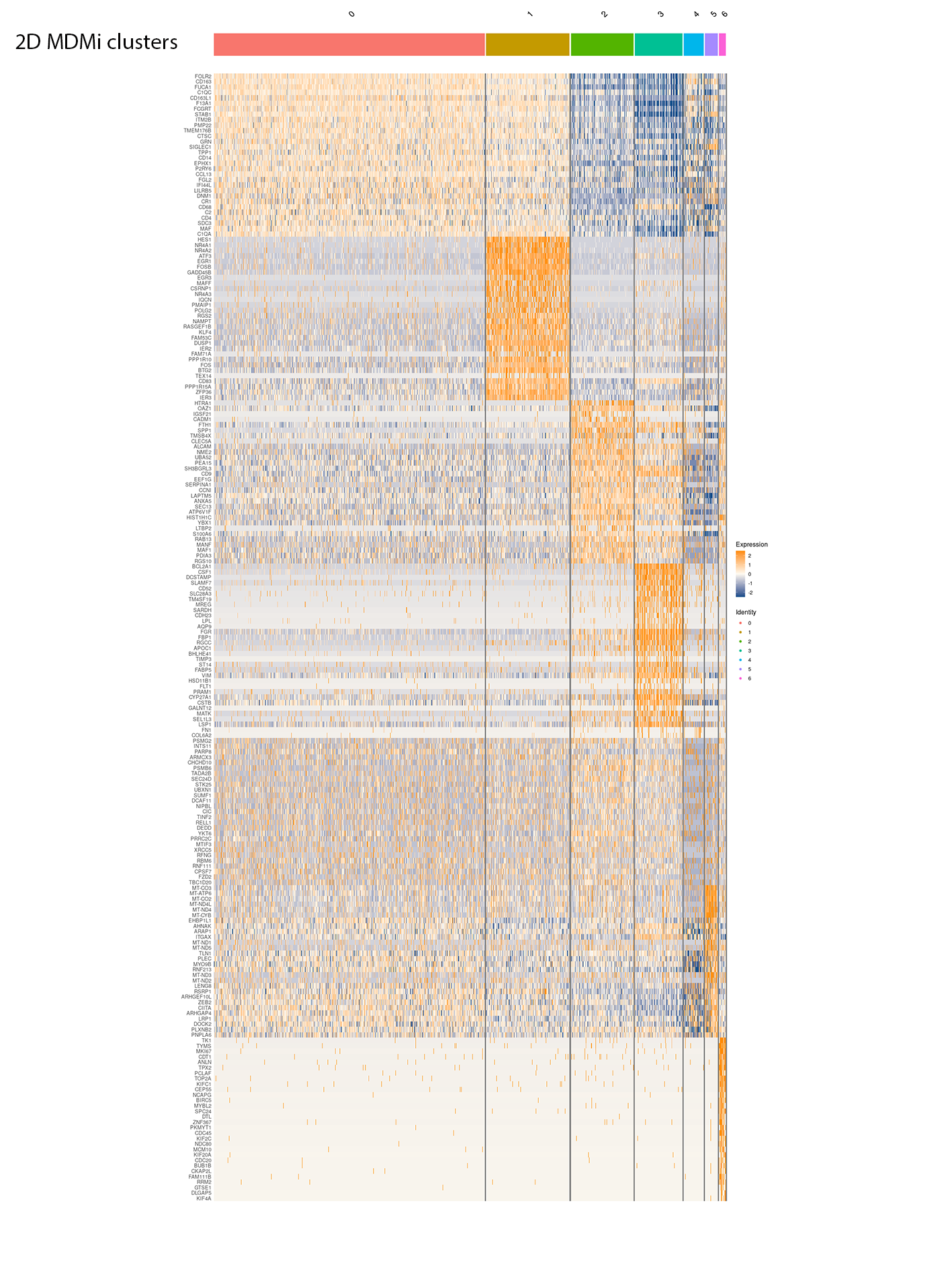

Supplement: Supplementary Figure 2 — Heatmap of normalized expression for selected differentially expressed genes in each cluster in 2D MDMi model. Range of log2 normalized counts are shown at right hand of heatmap. Blue indicates low expression; and orange indicates high expression. [file Image_2.tif]

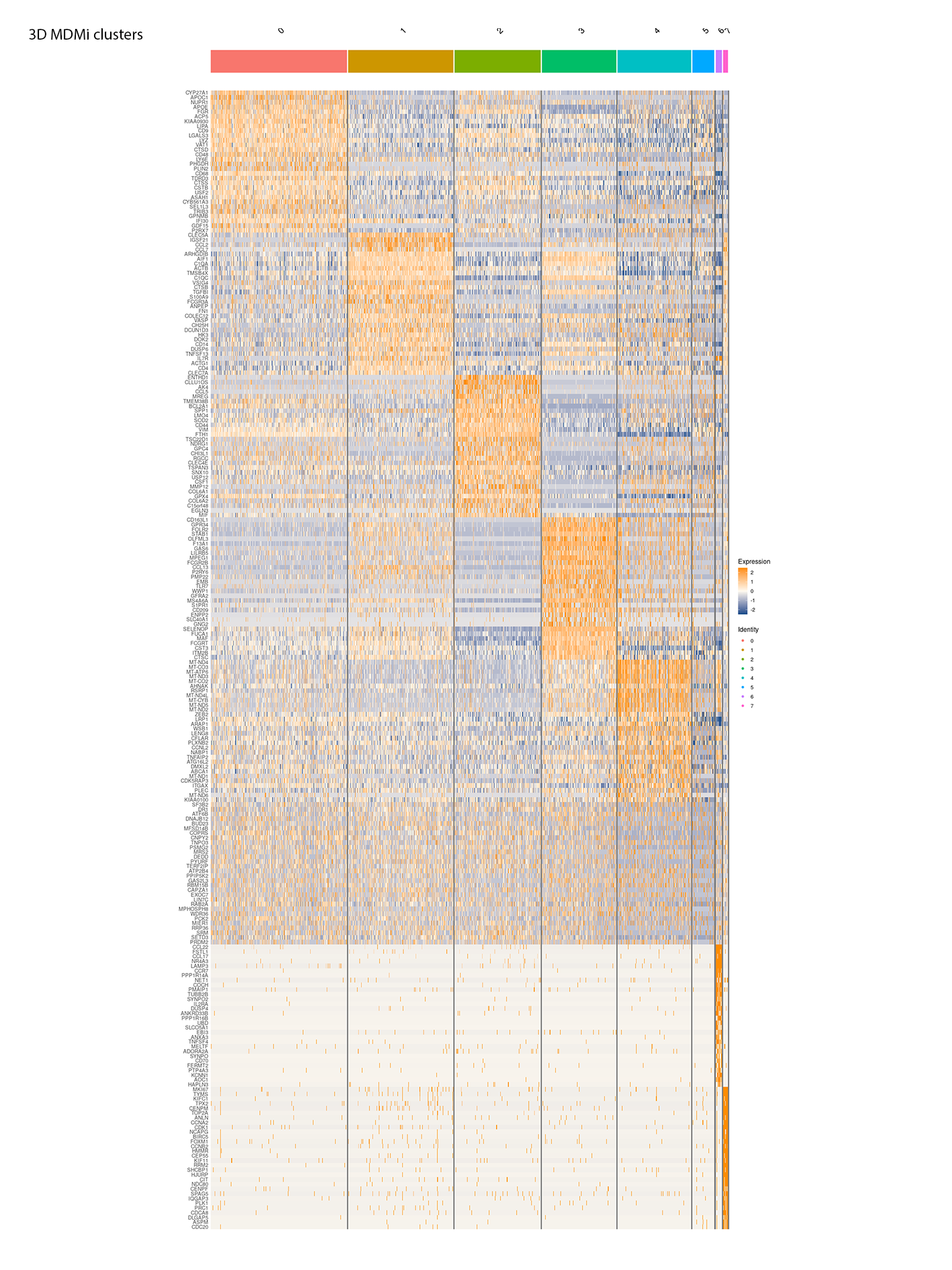

Supplement: Supplementary Figure 3 — Heatmap of normalized expression for selected differentially expressed genes in each cluster in 3D MDMi model. Range of log2 normalized counts are shown at right hand of heatmap. Blue indicates low expression; and orange indicates high expression. [file Image_3.tif]

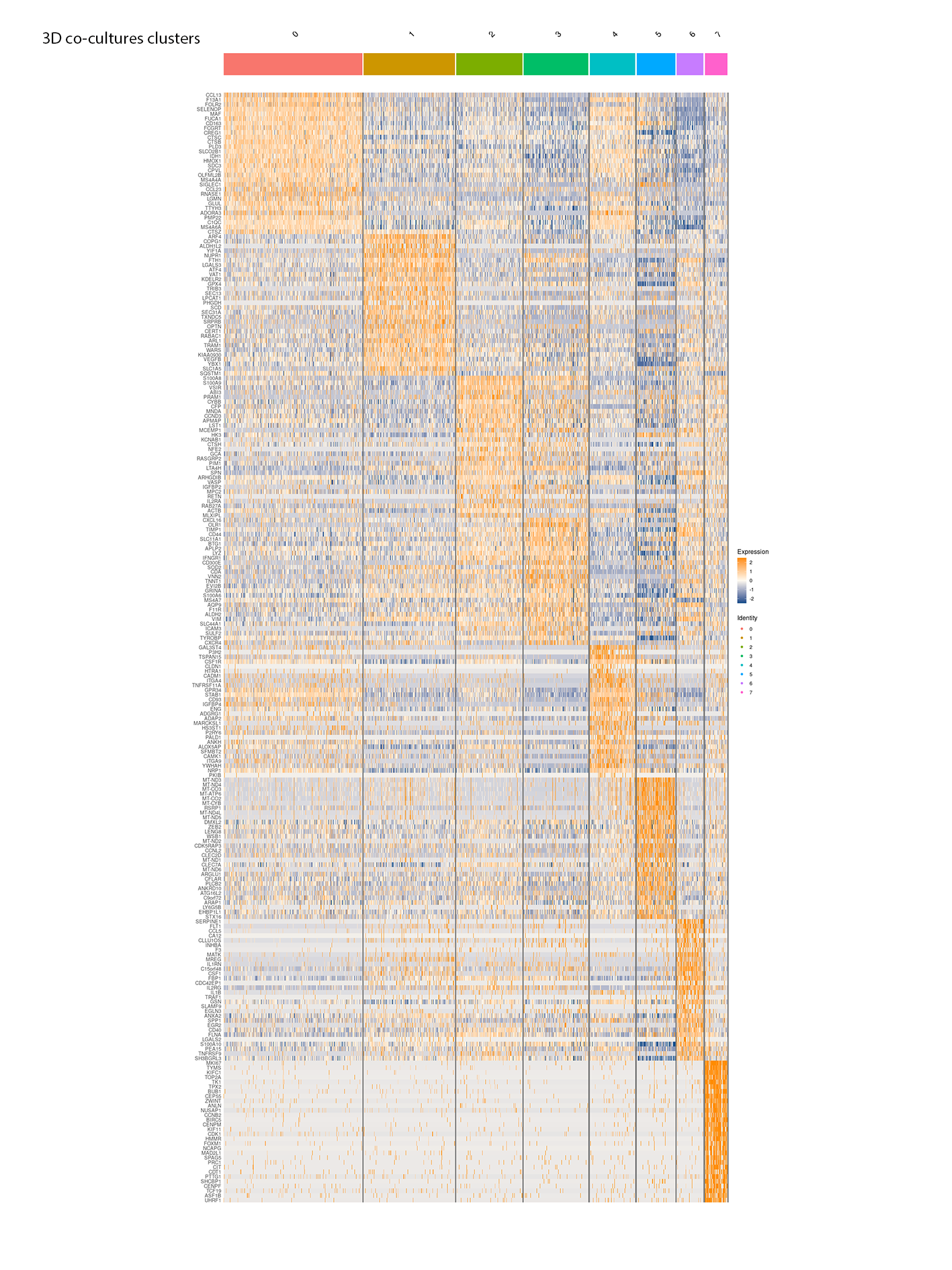

Supplement: Supplementary Figure 4 — Heatmap of normalized expression for selected differentially expressed genes in each cluster in 3D co-culture. Range of log2 normalized counts are shown at right hand of heatmap. Blue indicates low expression; and orange indicates high expression. [file Image_4.tif]

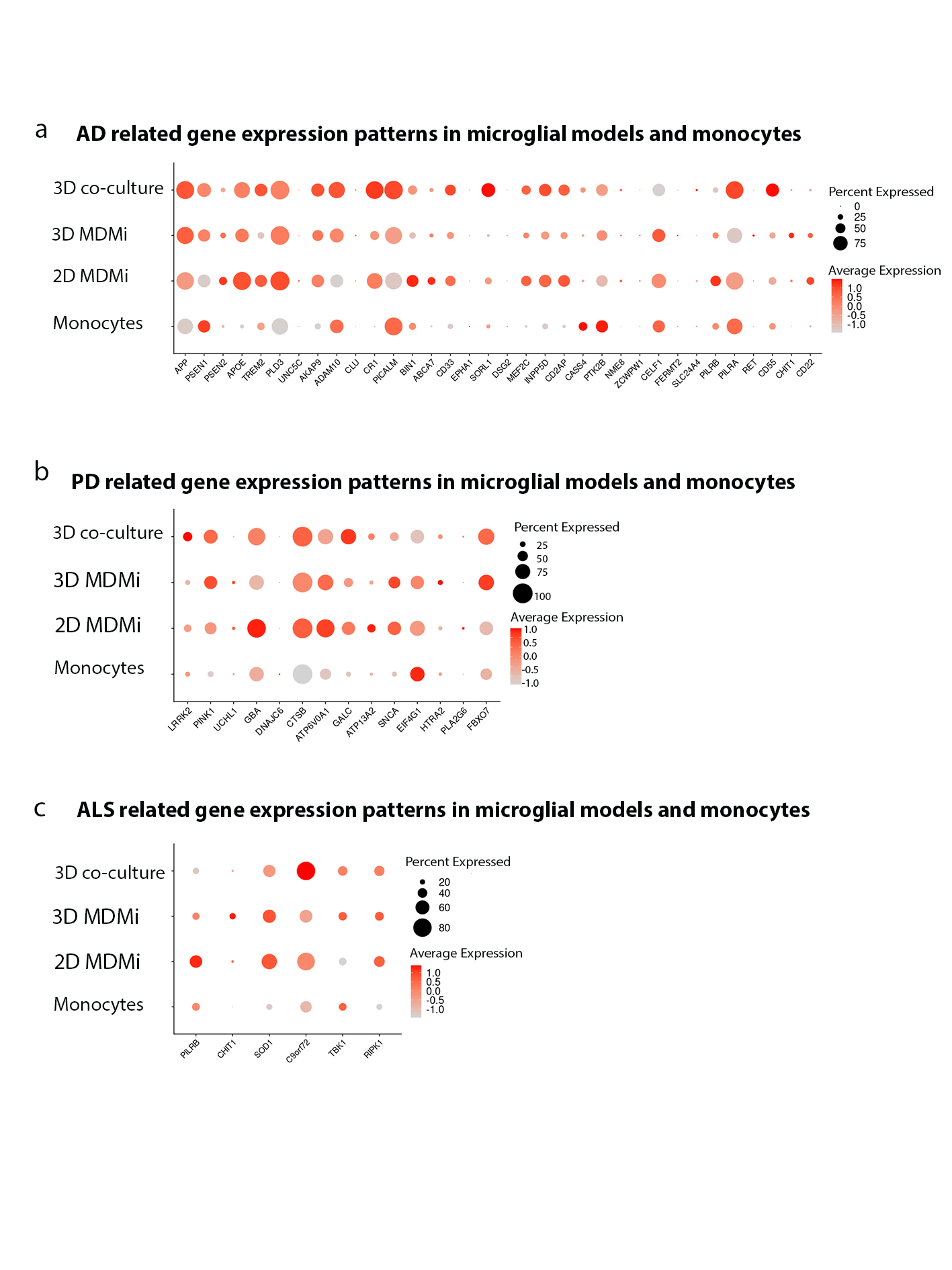

Supplement: Supplementary Figure 5 — Comparative analysis of microglial disease related genes in healthy 2D, 3D, and 3D co-culture models. (a) Dot plots illustrate AD related gene expression patterns in microglial models and monocytes, (b) dot plots illustrate PD related gene expression patterns in microglial models and monocytes, (c) dot plots illustrate ALS related gene expression patterns in microglial models and monocytes. Dot size indicating the percentage of cells expressing a given gene and color intensity reflecting the average expression level. [file Image_5.jpeg]

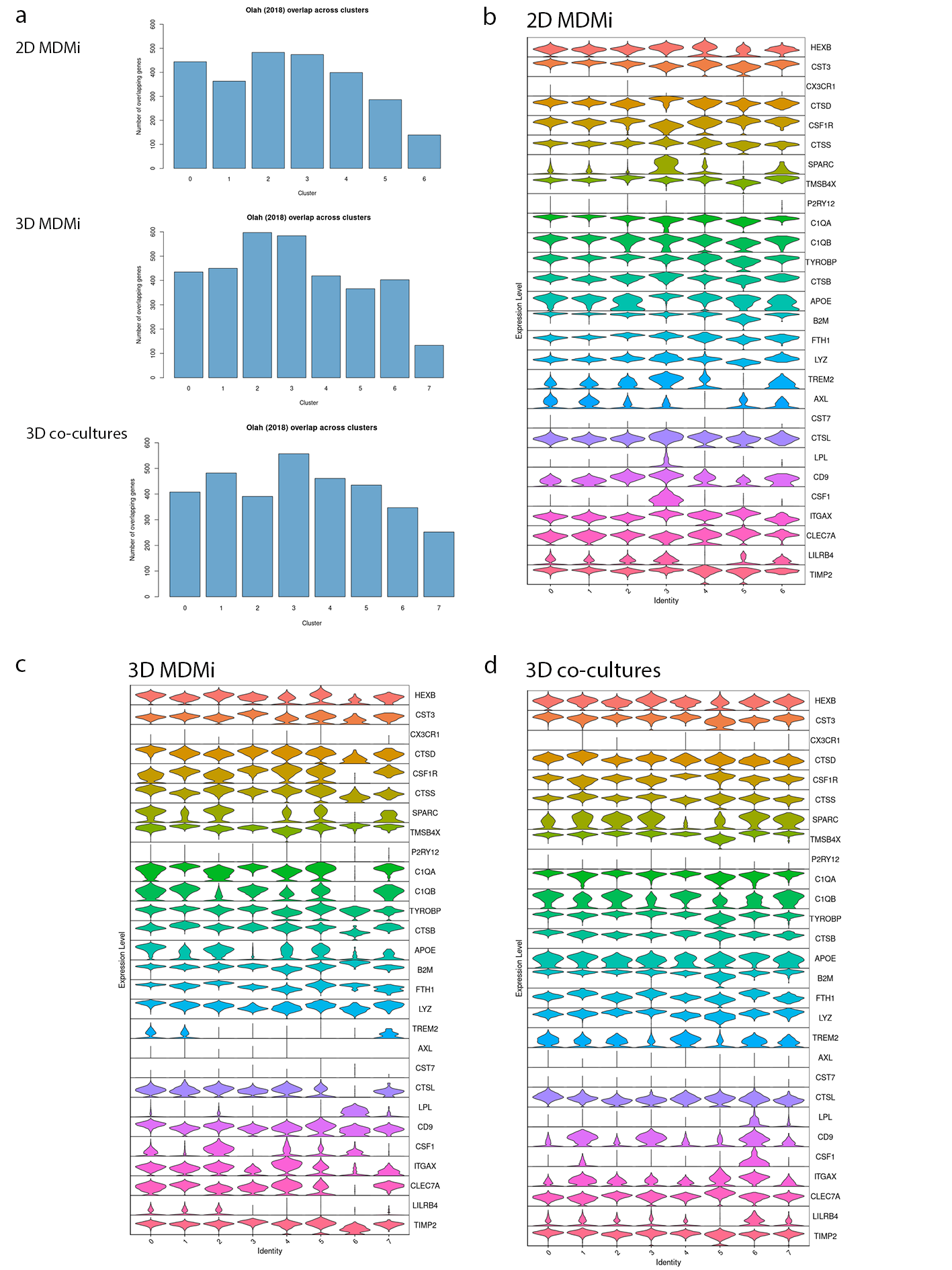

Supplement: Supplementary Figure 6 — Human microglial signature overlap and marker gene expression across MDMi culture conditions. (a) Bar plots showing the number of genes overlapping the human microglial transcriptional signature defined by Olah et al. (2020) across clusters in 2D MDMi, 3D MDMi, and 3D MDMi co-culture conditions. (b–d) Violin plots showing expression of selected microglial marker genes across clusters in 2D MDMi (b), 3D MDMi (c), and 3D co-culture (d) conditions. Genes are ordered from top to bottom according to canonical microglial identity, homeostatic, and activation-associated DAM genes. Each violin represents the distribution of normalized expression levels within a cluster. [file Image_6.tif]
